# Targeting *Fusobacterium nucleatum* through chemical modifications of host-derived transfer RNA fragments

**DOI:** 10.1038/s41396-023-01398-w

**Published:** 2023-04-01

**Authors:** Mengdi Yang, Pu-Ting Dong, Lujia Cen, Wenyuan Shi, Xuesong He, Jiahe Li

**Affiliations:** 1 Department of Bioengineering, Northeastern University, Boston, MA 02115 USA; 2 Department of Microbiology, The Forsyth Institute, Cambridge, MA 02142 USA; 3 Department of Oral Medicine, Infection and Immunity, Harvard School of Dental Medicine, Boston, MA 02115 USA; 4 Department of Biomedical Engineering, College of Engineering and School of Medicine, University of Michigan, Ann Arbor, MI 48109-5622 USA

**Keywords:** Antibiotics, Applied microbiology

## Abstract

Host mucosal barriers possess an arsenal of defense molecules to maintain host-microbe homeostasis such as antimicrobial peptides and immunoglobulins. In addition to these well-established defense molecules, we recently reported small RNAs (sRNAs)-mediated interactions between human oral keratinocytes and *Fusobacterium nucleatum (Fn)*, an oral pathobiont with increasing implications in extra-oral diseases. Specifically, upon *Fn* infection, oral keratinocytes released *Fn*-targeting tRNA-derived sRNAs (tsRNAs), an emerging class of noncoding sRNAs with gene regulatory functions. To explore potential antimicrobial activities of tsRNAs, we chemically modify the nucleotides of the *Fn*-targeting tsRNAs and demonstrate that the resultant tsRNA derivatives, termed MOD-tsRNAs, exhibit growth inhibitory effect against various *Fn* type strains and clinical tumor isolates without any delivery vehicle in the nanomolar concentration range. In contrast, the same MOD-tsRNAs do not inhibit other representative oral bacteria. Further mechanistic studies uncover the ribosome-targeting functions of MOD-tsRNAs in inhibiting *Fn*. Taken together, our work provides an engineering approach to targeting pathobionts through co-opting host-derived extracellular tsRNAs.

## Introduction

Host mucosal surfaces are highly specialized and possess a complex array of innate and adaptive immunity [1, 2]. They provide the first line of protection against infectious agents by initiating protective responses to potential pathogens. Furthermore, the symbiotic relationship of the hundreds of microbial species with the host requires a fine-tuned response at the mucosal surface that prevents overgrowth of opportunistic pathogens, while sparing beneficial microbes [2]. As a result, multiple innate and adaptive immune responses involving antimicrobial peptides, complement and immunoglobulins have evolved to maintain the delicate balance between the host and associated microbiomes [3–5]. In addition to these well-established systems, recent studies have begun to shed light on the roles of host-derived small RNAs (sRNAs) that contribute to the maintenance of host-microbial homeostasis through cross-kingdom gene modulation [6, 7]. For instance, eukaryotic cells secrete certain sRNAs (e.g., microRNAs) into extracellular environments, either encased in extracellular vehicles (EVs) or in an EV-free mode. These sRNAs target distantly related organisms and exert regulatory functions in a cross-kingdom fashion [8–10]. While extracellular miRNAs are employed by plants and vertebrate animals as a defense mechanism in the context of plant-pathogen and host-gut microbiota interactions [11–13], an emerging class of host-derived sRNA, named transfer RNA-derived small RNA (tsRNA), was recently identified to play a role in the host-bacteria interactions [14]. tsRNAs were originally identified to regulate gene expression inside eukaryotes in a cell autonomous manner [15]. However, accumulating evidence indicates that certain tsRNA species are produced and secreted by host cells under various physiological and pathological conditions, some of which have been proposed to serve as disease biomarkers [16]. In addition to these established roles, host-derived tsRNAs were recently implicated in the cross-kingdom interactions between human oral epithelial cells and *Fusobacterium nucleatum* (hereinafter *Fn*) [14].

Using a Normal Oral Keratinocyte-Spontaneously Immortalized (NOKSI)-*Fn* in vitro host-microbial interacting system, we demonstrated that when challenged with *Fn*, NOKSI cells released specific exosome-borne tsRNAs (tsRNA-000794 and tsRNA-020498) [14]. Furthermore, these two tsRNAs display selective antimicrobial activity—chemically synthesized tsRNA-000794 and tsRNA-020498 mimics, but not the scramble control inhibited the growth of *Fn*, while sparing *Streptococcus mitis (Sm)*, a health-associated Gram-positive oral bacterium. Meanwhile, these two host-derived tsRNAs can be readily detected in salivary exosomes from healthy human subjects [14], suggesting their potential role in targeted microbial modulation to help maintain host-microbial homeostasis. As a key oral pathobiont, *Fn* has garnered renewed attention in recent years. Specifically, in addition to being a bridging bacterium of dental plaque and its roles in periodontitis, *Fn* exhibits tolerance to oxygen to some extent, and can act as an oxygen sink to facilitate the growth of more strict anaerobes such as *Porphyromonas gingivalis* (*Pg*) [17]. Moreover, it has been postulated that *Fn* can be disseminated systemically from oral cavity to other organs contributing to extra-oral diseases such as adverse pregnancy outcomes, colorectal cancer, Alzheimer’s disease and various other diseases [18–20]. Our prior data suggested a new research avenue to repurpose host sRNAs for technology development and translational applications to achieve targeted depletion of disease-associated bacteria. However, despite the observed specificities at the sequence and species levels, excess synthetic tsRNA mimics (in the micromolar range) were required to inhibit *Fn*. This poses a formidable challenge to co-opting host-derived tsRNAs as a potential antimicrobial agent targeting *Fn*. Furthermore, a mechanistic understanding of tsRNA-mediated growth inhibition is still lacking.

**Fig. 1:**
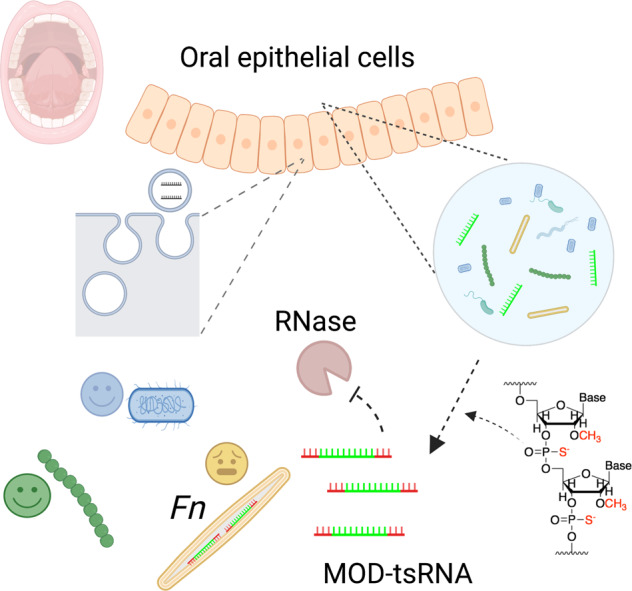
Targeting *Fn* through chemical modifications of host-derived tsRNAs. Certain host-derived tsRNAs from epithelial cells inhibit opportunistic pathogens in an inter-kingdom fashion at the mucosal interface. The figure is created by BioRender.

In the present work, to address the limitations, we drew on the power of rapid advances in chemically modified RNAs towards development of powerful genetic tools and therapeutic reagents, including FDA-approved small interfering RNA-based drugs [21], messenger RNA vaccines [22], and guide RNA for genome editing by Clustered Regularly Interspaced Short Palindromic Repeats (CRISPR) [23, 24]. Specifically, we adapted a similar chemical modification strategy to generate two modified tsRNAs (referred to as MOD(OMe)-tsRNAs in this study) matching the exact sequences of tsRNA-000794 and tsRNA-020498, which resulted in nearly 1000-fold increase in the efficacy of inhibiting *Fn* growth while preserving the sequence and species specificities (Fig. 1). We then demonstrated the uptake of MOD(OMe)-tsRNAs by multiple *Fn* strains of oral and gastrointestinal origins. Furthermore, RNA-seq analysis, tsRNA pull-down assay and Raman spectroscopy provided evidence that MOD(OMe)-tsRNAs likely inhibit *Fn* through a mechanism reminiscent of the mode of action for ribosome-targeting antimicrobials. In summary, our work exemplifies interdisciplinary approaches to understanding and harnessing host-derived tsRNAs to target oral pathobionts in a sequence- and species-specific fashion. Moreover, the study provides insight into the potential roles of host-derived tsRNAs in regulating bacterial physiology and host-microbial interactions.

## Materials and methods

Common chemicals, bacterial culture and standard assays are provided in SI Materials and Methods.

### Growth inhibition assays

Chemically synthesized tsRNAs and scrambled RNA control were reconstituted in sterile 1xPBS to obtain 500 μM stock concentrations stored at −80 °C. 10 μL different chemical modified tsRNA were seeded into 96-well plates with 2-fold serially diluted concentrations ranging from 5120 nM to 2 nM. The bacteria were diluted to OD_600_ = 0.01, and 90 μL diluted bacteria were added into individual wells of 96-well plates. The final concentrations were in the range of 1–512 nM. 10 μL of sterile 1xPBS was mixed with 90 μL diluted bacteria as an untreated control. Plates were incubated anaerobically at 37 °C, and OD_600_ was measured with a microplate reader at 0 h and 48 h to measure the outgrowth. For the CFU assay, the bacteria were grown to an OD_600_ of 0.1 in 1 mL growth media under anaerobic conditions, and then treated with 512 nM MOD(OMe)-tsRNA. Treated bacteria were washed to remove free tsRNAs at indicated times, and 10-fold serially diluted to enumerate CFU. Each growth inhibition assay was performed in three technical replicates and three biological repeats.

### tsRNA stability test and qPCR quantification

A mixture of three MOD-tsRNAs and naturally occurring tsRNAs were added to pre-reduced Columbia Broth. 100 μl sample was frozen immediately by liquid nitrogen as the start point (0 h) and stored at −80 °C for the next step. Samples were incubated at 37 °C for 3 h, 6 h, and overnight before snap freezing. The collected samples were treated with 20 μg ml^−1^ of proteinase K at 50 °C for 30 min and then 1 mM EDTA and PMSF were added to the samples and incubated at 95 °C for 10 min to inactivate proteinase K. tsRNAs were reverse transcribed to cDNA with a HiFiScript cDNA Synthesis Kit (CoWin BioSciences, Cambridge, MA, USA) by stem-loop primers. The detailed primer sequences and qPCR conditions are provided in SI Materials and Methods and Table S3. cDNA was amplified and quantified by a QuantStudio 3 Real-Time PCR System (ThermoFisher, Waltham, MA, USA).

### Fluorescence microscopy of Cy3-tsRNA uptake in bacteria

Cy3 labeled tsRNA was reconstituted in 10 mM Tris-HCI containing 0.1 mM EDTA for imaging. Overnight-grown bacteria were diluted to an OD_600_ of 0.1 and treated with 128 nM of 3’ Cy3 labeled tsRNA-000794, tsRNA-020498, and scrambled RNA control overnight (15–20 h) in an anaerobic chamber. Labeled bacteria were then washed with 0.9% NaCl for three times under a centrifuge speed of 17,000 x *g* for 10 min. Washed samples were then sandwiched between a cover glass and poly-L-lysine coated cover slide. Samples were then immediately imaged by a ZEISS LSM 800 confocal microscope with a fast Airyscan detector (with 120 nm lateral resolution and 350 nm axial resolution). To ensure the image quality, we utilized a 63x Plan-Apochromat NA1.4 oil immersion objective. Samples were excited at a wavelength of 514 nm with a 10% power and detected in the range of 550–600 nm. To quantify the fluorescence intensity from the same sample patch, dynamic range was adjusted to be the same under a channel-mode confocal modality. To have a clear visualization of tsRNA-Cy3 incorporation at subcellular level, super-resolution by Airyscan was achieved at a gain of 800 V. Images were visualized and analyzed by FiJi (NIH) and OriginLab (OriginLab Corporation).

### RNA sequencing and data analysis

RNA sample quality was assessed using a Nanodrop (ThermoFisher) and Agilent 5400 (Agilent Technologies, Santa Clara, CA, USA). Prokaryotic mRNA sequencing was performed using the NovaSeq PE150 platform (Illumina, San Diego, CA, USA) at the Novogen facility (Sacramento, CA, USA). The library was prepared by a Ribo-Zero protocol (250 − 300 bp insert strand specific library with rRNA removal using NEB Ribo-Zero Magnetic Kit). Paired-end sequencing produced 150 bp reads, to a depth of ~2 G output per sample. Sequences were mapped to a reference genome, *Fusobacterium nucleatum* ATCC 23726 (GenBank accession: CP028109) using a Bowtie2 pipeline adjusted for paired-end sequencing. Differential gene expression was analyzed using the DEseq2 pipeline in Rstudio as previously described [25]. The total mapping rates with respect to the annotated genome for *Fn* 23726 were >98%. The false discovery rate (FDR) was set to 5% and genes with a log_2_FoldChange of >1 or < −1 and a *p*-adjusted value (*p-*adj) < 0.001 were considered significant. All reported data are representative of three biological replicates.

### Statistical analysis

All statistical analyses were performed using GraphPad Prism 9 (San Diego, CA, USA). Data were analyzed with the student t-test, one-way or two-way analysis of variance (ANOVA) followed by *Bonferroni* test for statistical significance.

## Results

### Chemical modifications of host-derived *Fn*-targeting tsRNAs enhance their inhibitory efficacy while maintaining the sequence and species specificities

**Fig. 2:**
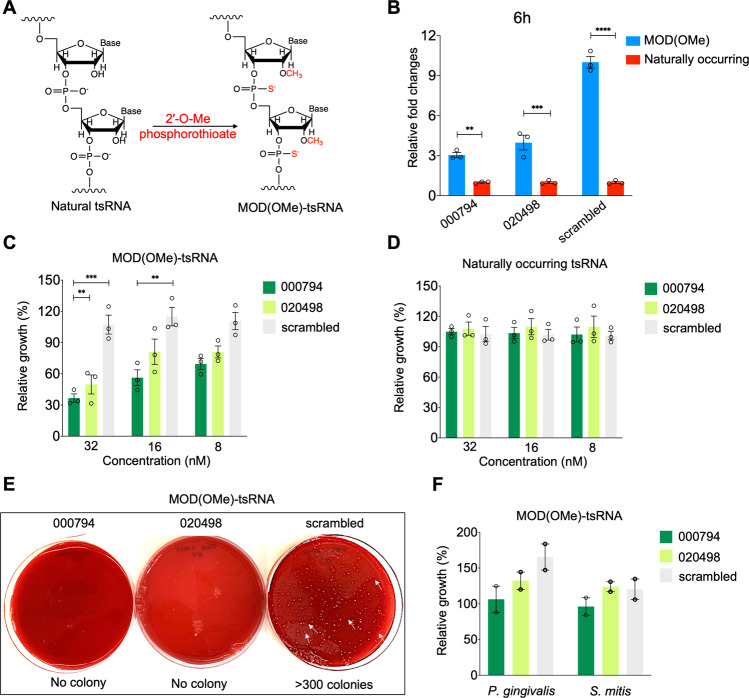
Chemically modified *Fn*-targeting tsRNAs confer superior growth inhibition of *Fn* in a sequence-, and species-dependent manner. **A** Incorporation of 2’-OMe phosphorothioate linkage in tsRNAs, referred to as MOD(OMe)-000794, MOD(OMe)-020498 and MOD(OMe)-scrambled RNA. **B** Improvement of tsRNA stability by chemical modifications in comparison to synthetic mimics of naturally occurring counterparts. Individual tsRNAs (1 nM) were incubated with Columbia broth anaerobically at 37 °C for 6 h, and intact tsRNAs were measured via quantitative real time PCR using a stem-loop method. Fold changes were normalized to the levels of naturally occurring tsRNAs, which are shown as “1” on the y axis. *n* = 3 technical replicates, *N* = 2 independent experiments. **C** Growth inhibition of *Fn* ATCC 23726 by MOD(OMe)-000794 and MOD(OMe)-020498, but not MOD(OMe)-scrambled RNA, at the nanomolar concentration ranges (*n* = 3, *N* = 3). **D** Naturally occurring tsRNAs failed to inhibit *Fn* ATCC 23726 at indicated concentrations (*n* = 3, *N* = 3). **E** Representative images showing the colony formation of *Fn* ATCC 23726 on agar plates after treatment with 512 nM MOD(OMe)-000794, MOD(OMe)-020498 or MOD(OMe)-scrambled RNA for 24 h. Bacteria were washed and recovered on nonselective Columbia broth sheep blood agar plates. Representative colonies are indicated by white arrows. **F** MOD(OMe)-000794 and MOD(OMe)-020498 at 512 nM exhibited no growth inhibition in two representative oral bacteria, a Gram-negative bacterium, *Porphyromonas gingivalis* ATCC 33277 (*P. gingivalis*) and a Gram-positive bacterium, *Streptococcus mitis* ATCC 6249 (*S. mitis*). (*n* = 2, *N* = 2). Data were analyzed by the two-way ANOVA followed by Dunnett’s Bonferroni multiple comparison tests. **p < 0.01, ***p < 0.001, ****p < 0.0001. Data are means ± SEM.

Host derived extracellular tsRNAs are typically encapsulated in extracellular vesicles or associated with proteins to confer tsRNAs stability in human saliva. In our earlier work, directly adding synthetic mimics of naturally occurring *Fn*-targeting tsRNAs inhibited the growth of *Fn* albeit with a low efficacy (the half maximal inhibitory concentration (IC_50_) is ~50 µM) [14], likely due to the susceptibility of naked RNA to nuclease-mediated degradation in bacterial culture. To increase the stability of tsRNAs, we explored two common RNA modifications (Table 1 and Fig. 2A): First, a 2’ methoxy group (2’-OMe) substituted the 2’ hydroxyl group of the ribose moiety of the three terminal nucleosides at both 5’ and 3’ ends [24]. Of note, 2’-OMe is prevalent in both prokaryotes and eukaryotes as a key post-transcriptional RNA modification for noncoding RNA species, among which piRNAs and miRNA bear a 2’-O-methylated nucleotide at the 3’ end [26–28]. Moreover, 2’-OMe has been frequently employed in modifying siRNAs for RNA interference and guide RNAs for CRISPR genome editing [21, 23, 24]. Second, two phosphodiester linkages were replaced with phosphorothioate (PS) bonds at both 5’ and 3’ termini, in which a non-bridging oxygen in the phosphodiester bond was substituted with a sulfur atom. While these two modification strategies have been widely used to confer nuclease resistance on guide RNA and siRNAs for genome editing and RNA interference [21, 24], it has yet to be confirmed whether 2’-OMe and PS modifications can also improve the stability and *Fn*-inhibiting efficacy of the naturally occurring tsRNAs that we previously identified. For the rest of this study, we will use MOD(OMe)−000794, MOD(OMe)-020498 and MOD(OMe)-scrambled to specify the chemically modified tsRNA-000794, tsRNA-020498 and the non-targeting scrambled RNA control, respectively. Using a stem-loop reverse transcription polymerase chain reaction (RT-PCR) assay, it was confirmed that MOD(OMe)-000794, MOD(OMe)-020498, and MOD(OMe)-scrambled displayed enhanced stability over that of the naturally occurring ones after 3, 6, and 24 h incubation in bacterial culture media (Fig. 2B, Fig. S1A). Furthermore, MOD(OMe)-tsRNAs were also shown to exhibit higher stability than that of the natural counterparts when treated with human saliva (Fig. S1B), suggesting potential translation using MOD(OMe)-tsRNAs over natural tsRNAs.

**Table 1:** List of chemically modified and naturally occurring tsRNAs.

RNA name	RNA Sequence (5′ to 3′)
MOD(OMe)-000794	C*C*GGCUAGCUCAGUCGGUAGAGCAUGA*G*A
MOD(OMe)-020498	G*G*GGGUAUAGCUCAGUGGGUAGAGC*A*U
MOD(OMe)-scrambled	G*G*ACGACAAGUUCGUGACGAGCGCAUC*U*G
tsRNA-000794	CCGGCUAGCUCAGUCGGUAGAGCAUGAG**A**
tsRNA-020498	GGGGGUAUAGCUCAGUGGGUAGAGCA**U**
scrambled	GGACGACAAGUUCGUGACGAGCGCAUCU**G**

(1) Nucleotides highlighted in bold have 2’-OMe substitution at the 2’-OH of RNA ribose as shown in Fig. 1A.

(2) * denotes phosphorothioate (PS) bonds, which are used in combination with 2’-OMe substitution.

Having validated the enhanced stability of modified tsRNAs over their naturally occurring counterparts, we further assessed whether MOD-tsRNAs exhibit increased efficacy of inhibition against *Fn*. It was found that MOD(OMe)-000794 and MOD(OMe)-020498 inhibited the growth of *Fn* ATCC 23726 in a dose-dependent manner, achieving an IC_50_ in the range of 16–32 nM (Fig. 2C). In comparison, at the same concentrations, naturally occurring tsRNAs did not affect the growth of *Fn* ATCC 23726 (Fig. 2D), which agreed with our earlier findings on the requirement of a micromolar concentration range to inhibit *Fn* by naturally tsRNAs [14]. In addition, MOD(OMe)-scrambled RNA control did not inhibit *Fn* ATCC 23726 even at 512 nM, indicating that the observed inhibition was dependent on the specific tsRNA sequence rather than chemical modifications (Fig. S2). Meanwhile, MOD(OMe)-000794 and MOD(OMe)-020498, but not MOD(OMe)-scrambled RNA control, also inhibited the growth of one additional *Fn* type strain, *Fn* ATCC 25586 (Fig. S3), albeit with reduced inhibition efficacy. These findings suggest possible differences in MOD-tsRNA susceptibility at the strain level, as both *Fn* ATCC 23726 and 25586 belong to *Fn* subsp. *nucleatum* [29].

Considering a ~1000-fold improvement in inhibition efficacy for MOD-tsRNAs over the naturally occurring tsRNA counterparts, we further asked whether *Fn* were able to regrow after the treatment with MOD-tsRNAs. To this end, we treated *Fn* ATCC 23726 with 512 nM of MOD(OMe)-000794, MOD(OMe)-020498, and MOD(OMe)-scrambled RNA control, respectively for 24 h, and recovered bacteria on non-selective Columbia broth blood agar to enumerate the colony-forming unit (CFU). 24 h treatment with MOD(OMe)-000794 or MOD(OMe)-020498 resulted in complete killing of *Fn* ATCC 23726 cells (Fig. 2E), approximately nine orders of magnitude of reduction in the CFU compared to that of MOD(OMe)-scrambled RNA control (Fig. S2A). Decrease in the CFU was further demonstrated in *Fn* ATCC 25586 (Fig. S4), after 24 h treatment with MOD(OMe)-000794 or MOD(OMe)-020498 but not MOD(OMe)-scrambled RNA control. These findings suggested highly potent killing of *Fn* by MOD-tsRNAs. To test whether bacteria can recover in the fresh liquid medium after MOD-tsRNAs were removed, only MOD(OMe)-scrambled RNA control and PBS pretreatment for 24 h showed bacterial re-growth while pretreatment with MOD(OMe)-000794 or MOD(OMe)-020498 for 24 h did not (Fig. S2B).

In addition to the sequence specificity, we tested whether MOD-tsRNAs displayed *Fn*-specific growth inhibition. To this end, we challenged two representative oral bacteria including Gram-negative *Porphyromonas gingivalis* ATCC 33277 (*Pg*), and Gram-positive *Streptococcus mitis* ATCC 6249 (*Sm*) (Fig. 2F), as well as *E. coli* K-12 (Fig. S5) with MOD(OMe)-000794, MOD(OMe)-020498 and MOD(OMe)-scrambled RNA control. Indeed, none of these three bacteria was inhibited by MOD(OMe)-tsRNAs at a concentration of 512 nM (>10-fold higher than the IC_50_ of MOD-tsRNAs for *Fn* ATCC 23726). Since the two naturally occurring tsRNAs were originally identified in human saliva and can also be secreted by human oral epithelial cells [14], we further tested whether MOD-tsRNAs affect the viability of host cells. It was shown that 512 nM of MOD(OMe)-000794 or −020498 did not affect the proliferation of immortalized human oral epithelial cells in comparison to the untreated or MOD(OMe)-scrambled RNA control (Fig. S6).

Motivated by the enhanced efficacy of tsRNA through partial chemical modifications at the three terminal nucleotides, we further explored and compared the efficacy of fully modified tsRNAs in inhibiting *Fn* growth with naturally occurring and partially modified sequences (Fig. S7A). Full modifications of RNA backbone completely abolished the efficacy (Fig. S7B), which underscores the degrees of modifications in dictating the anti-*Fn* properties of tsRNAs. These findings are in line with prior studies in RNA interference [30] and CRISPR genome editing [23, 24], which demonstrated that extensive chemical modifications compromised the activities of siRNA and guide RNA likely through altering the secondary structure of RNA, affecting interaction between RNA and target proteins, or inducing nonspecific interactions with cellular components. Altogether, our findings highlighted the importance of the degrees of RNA modifications towards enhanced stability and efficacy of tsRNAs.

### Chemically modified tsRNAs inhibit *Fn* clinical tumor isolates

**Fig. 3:**
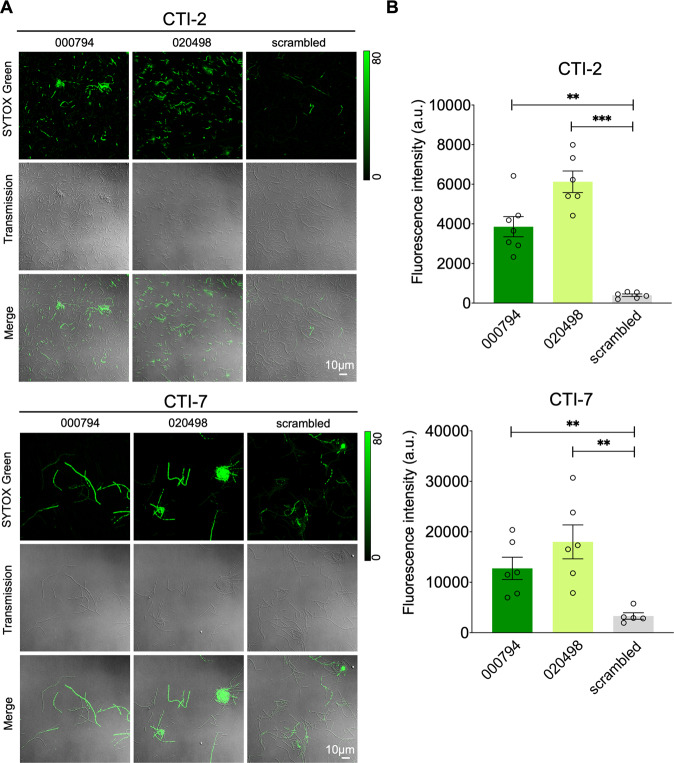
Chemically modified tsRNAs inhibit *Fn* clinical tumor isolates. **A** *Fn* CTI-2 and CTI-7 were treated with 500 nM MOD(OMe)-000794, MOD(OMe)-020498, and MOD(OMe)-scrambled for 48 h followed by SYTOX Green staining. **B** SYTOX Green quantification was carried out by normalizing raw integrated fluorescence intensity to the areas of randomly picked bacteria, which take into account both SYTOX Green-positive and -negative ones in the field of view. Data were representative of two independent experiments and analyzed by the unpaired t-test. **p < 0.01, ***p < 0.001.

*Fn* has garnered increasing attention due to its implications in colorectal cancer (CRC) [31, 32]. In addition to two type strains (*Fn* ATCC 23726 and 25586), we further tested *Fn* clinical tumor isolates (CTI) from CRC by challenging them with the same MOD-tsRNAs. We chose CTI-2 and CTI-7 due to their distinct adhesion characteristics for CRC cells. Specifically, the interaction between D-galactose-b(1-3)-N-acetyl-D-galactosamine (Gal-GalNAc) from cancer cells and a surface protein, Fap2, from *Fn* has been shown to promote the enrichment of fusobacteria in CRC. While CTI-2 expresses Fap2 that mediates the adhesion of *Fn* to CRC epithelial cells overexpressing Gal-GalNAc, CTI-7 is *fap2*-deficient and does not efficiently attach to cancer cells [33]. Unlike type strains, however, CTI-2 and CTI-7 grow at a slower rate under conditions used in this study, and tend to form aggregates, rendering it less accurate to quantify the growth rates by measuring the absorbance. Nonetheless, after 48 h treatment, the absorbance measurement showed that MOD(OMe)-000794 and −020498 inhibited the growth of CTI-2 and CTI-7 while MOD(OMe)-scrambled RNA control did not (Fig. S8). To further corroborate the absorbance measurement, we selected a cell viability dye, SYTOX Green, which emits an intense fluorescence in membrane-compromised bacteria due to its strong binding affinity to nucleic acids. We reasoned that the SYTOX Green dye offers an alternative yet more sensitive way to detect the response of *Fn* isolates than that of absorbance measurement. The results showed that MOD(OMe)-tsRNAs treatment induced significant killing of CTI-2 and CTI-7 after 48 h incubation compared to that of the scramble control (Fig. 3A, B). Similar to *Fn* ATCC 25586, however, higher concentrations (500 nM) of MOD(OMe)-000794 and −020498 were required to achieve significant cell death in CTI-2 and CTI-7, which may reflect subspecies or strain variation in resistance to tsRNAs.

### Internalization of *Fn*-targeting tsRNAs by *Fn* but not *Sm* or *Pg*

**Fig. 4:**
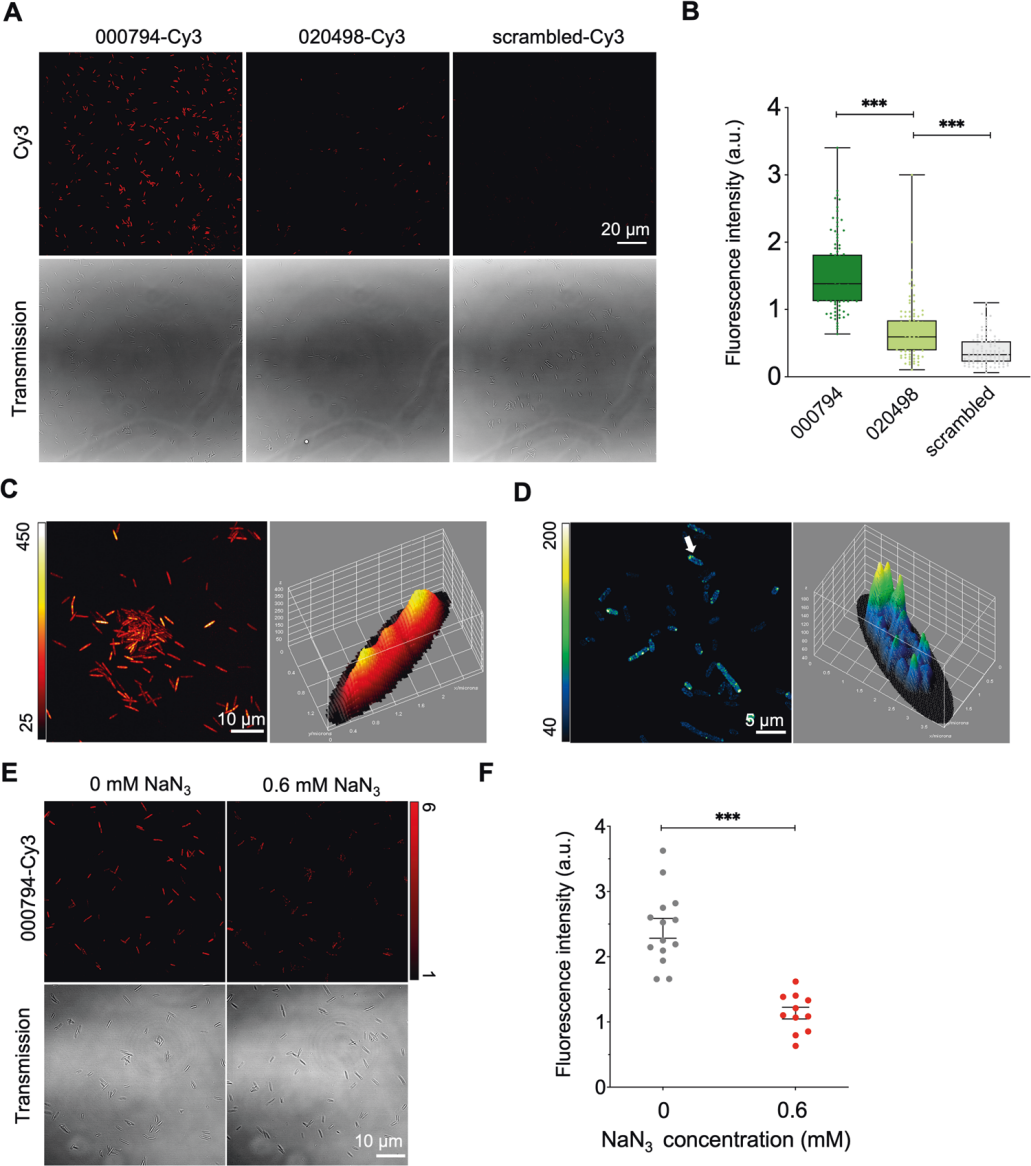
Internalization of host-derived tsRNAs by *Fn*. **A** Treatment of *Fn* ATCC 23726 with 128 nM tsRNA-000794-Cy3, tsRNA-020498-Cy3 or scrambled RNA-Cy3, and subsequent visualization by fluorescence microscopy. **B** Quantification of fluorescence intensity by normalizing raw integrated fluorescence intensity to the areas of bacteria as shown in **A**. Box-and-whisker plots: median, horizontal line; box range, percentile 25, 75. Data was determined by the unpaired t-test (***p < 0.001). Differential localization of tsRNA-000794-Cy3 in (**C**) *Fn* ATCC 23726 and (**D**) *Pg* ATCC 33277. The images were acquired by Airyscan confocal microscopy, and a randomly picked bacterium was projected with the height (z-axis) indicating the levels of tsRNA-000794-Cy3 (on the right-hand side). Images are representative of three biological replicates. **E** The uptake of tsRNA-000794-Cy3 in the absence or presence of sodium azide (metabolic energy inhibitor). **F** Quantification of fluorescence intensity for tsRNA-000794-Cy3 in randomly selected *Fn* bacteria. Statistical significance was determined by the unpaired t-test (***p < 0.001).

Prior studies in host-gut microbiome and plant-fungi interactions demonstrated that host-derived sRNAs can enter bacteria and fungi to modulate their physiology [11–13]. Given the inhibitory ability of two tsRNAs against *Fn*, we next asked whether the *Fn*-targeting tsRNAs also entered *Fn* to exert the growth inhibition. We fluorescently labeled the tsRNAs with Cy3 at the 3’ end and treated *Fn* ATCC 23726 with tsRNA-000794-Cy3, tsRNA-020498-Cy3, or the scrambled-RNA-Cy3. After overnight incubation, *Fn* ATCC 23726 displayed higher fluorescence signal from the treatment of tsRNA-000794-Cy3 than that of tsRNA-020498-Cy3, with the scrambled-RNA-Cy3 showing the lowest signal retention (Fig. 4A, B). These findings agreed with the slightly higher growth inhibition of *Fn* 23726 by MOD(OMe)-000794 than that of MOD(OMe)-020498 (Fig. 2C). To further confirm the intracellular uptake, we examined the localization of fluorescently labeled tsRNAs in *Fn* ATCC 23726 through the super-resolution fluorescence microscopy with an Airyscan detector, an optical technique that allows us to detect nanoscale morphological features at a higher resolution than that of conventional confocal microscopy [34]. It was found that the majority of tsRNA-000794-Cy3 accumulated in the cytoplasm of *Fn* ATCC 23726 (Fig. 4C), and a similar uptake was observed in *Fn* ATCC 25586 (Fig. S9), suggesting that the two *Fn* strains likely shared the same mechanism in internalizing tsRNAs. Given the higher uptake of tsRNA-000794-Cy3 than tsRNA-020498-Cy3, we subsequently focused on characterizing tsRNA-000794-Cy3 intake in the clinical tumor isolates. Similar to the findings in *Fn* ATCC 23726, Cy3-labeled tsRNAs were also localized in the cytoplasm of two *Fn* clinical tumor isolates after 24 h incubation (Fig. S10), which further suggested that MOD(OMe)-tsRNAs entered *Fn*, regardless of their origins, to mediate the growth inhibition (Figs. 1C, 2B). We next tested whether tsRNAs were internalized through passive diffusion or active transport. Sodium azide was employed here because it has been shown to repress the ATPase in *E. coli* across the membrane under anaerobic conditions [35]. Indeed, it was found that the internalization of tsRNA-000794-Cy3 was markedly reduced in the presence of 0.6 mM sodium azide (Fig. 4E, F), while this concentration minimally affected the growth of *Fn* ATCC 23726 (Fig. S11). Therefore, the sodium azide inhibition study suggested that the uptake of tsRNAs is dependent on an active transport mechanism rather than passive diffusion. To confirm that the observed intake was not due to specific properties of the linked dye molecule, we next substituted Cy3 with Alexa-488, a structurally and spectrally different dye molecule. Similarly, we observed enhanced uptake of tsRNA-000794-Alexa 488 and tsRNA-020498-Alexa 488 compared to the scrambled RNA-Alexa 488 (Fig. S12A). Additionally, two Alexa 488 conjugated tsRNAs but not the scrambled RNA could inhibit the growth of *Fn* ATCC 23726 (Fig. S12B, C). Altogether, our results showed that (1) tsRNAs can be internalized by *Fn*; and (2) 3’ end labeling of tsRNA with a fluorescent dye did not significantly interfere with its inhibitory effect against *Fn*.

Since the two MOD-tsRNAs can specifically kill *Fn* but spare other oral bacteria such as *Pg* and *Sm*, we further asked whether the selective killing by MOD-tsRNAs against *Fn* can be attributed to different uptake patterns in *Pg* and *Sm*. In contrast to their cytoplasmic accumulation in *Fn* ATCC 23726, *Fn* ATCC 25586 and clinical tumor isolates, the same MOD-tsRNA-Cy3 was primarily located at the periphery of *Pg* and *Sm* as evidenced by the Airyscan confocal microscopy imaging (Fig. 4D, Fig. S13). While the differential uptake patterns for tsRNA-Cy3 between *Fn*, *Pg*, and *Sm* may be implicated in the targeted growth inhibition against tsRNAs in *Fn*, it remains to be determined how MOD-tsRNAs affected the growth of *Fn*.

### Global RNA profiles show MOD-tsRNAs targeting protein translation

To further investigate the mechanisms of MOD-tsRNA-mediated growth inhibition, we performed bacterial RNA sequencing (RNAseq) to compare transcriptomic differences between MOD(OMe)-000794 and MOD(OMe)-scrambled control treated *Fn* ATCC 23726. It is critical to optimize both concentrations and treatment duration such that the transcriptomics does not merely reflect cell death-associated gene expression changes. In addition, we reasoned that if *Fn* ATCC 23726 is treated with lower concentrations or shorter duration than the optimal conditions, there may not be significant transcriptomic changes to infer the targets and functions of MOD-tsRNAs. For these reasons, global transcriptome studies of antimicrobial responses generally use either sub-inhibitory concentrations of the inhibitors of interest for a relatively long duration or analyze transcriptome profiles soon after exposure to a lethal concentration, with each approach having its advantages and disadvantages [36]. Here, we opted for RNA-seq at an early time point after treating *Fn* ATCC 23726 with inhibitory doses of MOD(OMe)-000794 or MOD(OMe)-scrambled RNA control and analyzed bacterial samples by RNAseq. Since we focused on the short-term response of *Fn* when exposed to a lethal concentration of MOD-tsRNA, a 10-time higher starting OD (OD_600_ = 0.2) than the viability assay was used to obtain enough bacteria for RNA extraction. However, it was difficult to rely on absorbance measurement at early time points to optimize the concentrations of MOD-tsRNAs that can induce detectable inhibition in the bacterial samples. To address the issues, the SYTOX Green viability dye was employed to monitor differences in cell viability when *Fn* ATCC 23726 were treated with MOD(OMe)-000794 or MOD(OMe)-scrambled at various concentrations within a short treatment period. Approximately ~10% reduction in cell viability for *Fn* ATCC 23726 was observed under the treatment condition at 500 nM MOD(OMe)-000794 for 5 h (Fig. S14), which corresponds to approximately one round of cell division for *Fn* according to our experiment and the literature [37, 38].

**Fig. 5:**
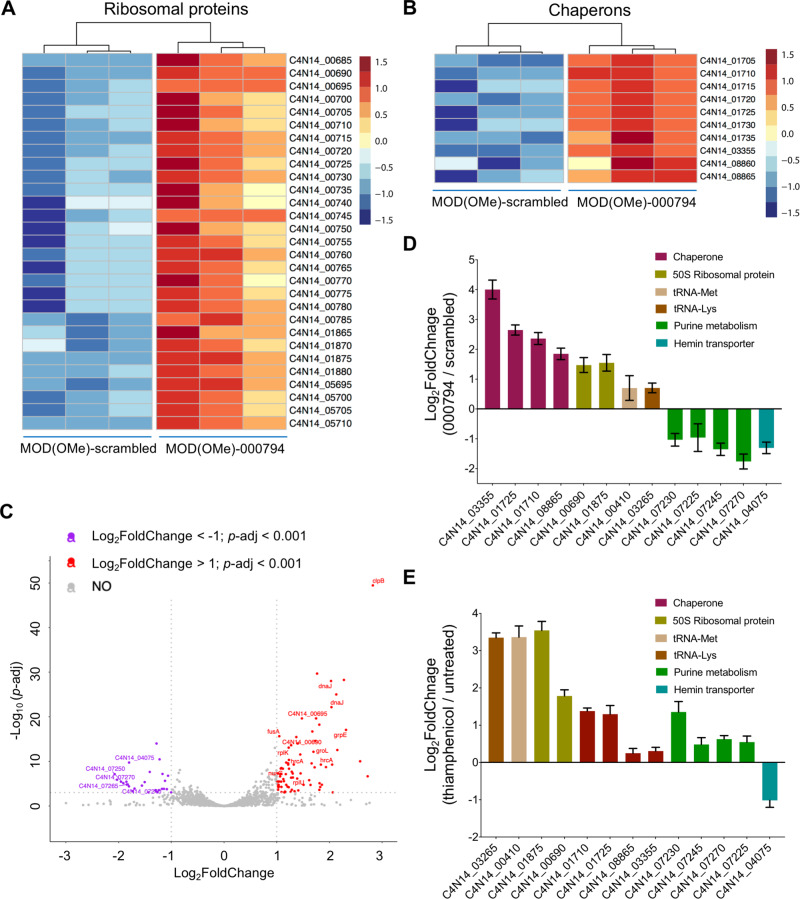
Global RNA profiles show MOD-tsRNAs targeting protein translation. Heatmap showing differentially expressed ribosomal protein-encoding genes (**A**) and chaperone-encoding genes (**B**) upon the indicated treatment conditions. Each heatmap includes triplicate RNA-seq samples for the indicated MOD(OMe)-000794 or MOD(OMe)-scrambled treatment. The coloring indicates log_2_FoldChange of the selected samples, while red and blue denote up- and down-regulation, respectively. The DESeq2 method (*p-*adj < = 0.05, |log2FoldChange | > = 0.0) was applied to generate the heatmap. *p*-adj refers to *p*-value adjusted. **C** Volcano plots showing transcriptomic changes of *Fn* ATCC 23726 in response to 500 nM MOD(OMe)-000794 relative to the MOD(OMe)-scrambled control RNA at 5 h. Shown in the plot are false discovery rate (FDR)-adjusted *p*-value (–log_10_*p*-adj, y-axis) and fold change (log_2_FoldChange, x-axis). Significantly differentially regulated genes are characterized by an absolute fold change >2 (down-regulated log2 < −1, up-regulated log_2_ > 1; vertical dashed line) and an FDR-adjusted *p* < 0.001 (–log_10_*p*-adj >3, horizontal dashed line). A full list of differentially expressed genes can be found in Dataset S2. **D** Validation of differentially expressed genes during MOD(OMe)-000794 treatment using RT-PCR. Relative gene expression (log_2_FoldChange) was normalized to 16 S reference gene by the 2^–∆∆Ct^ method, relative to the MOD(OMe)-scrambled control. **E** RT-PCR quantification of differentially expressed genes in *Fn* ATCC 23726 treated with 1 μg mL^−1^ thiamphenicol relative to the untreated control. **D**, **E** are means ± SEM of three independent experiments. Please refer to Tables S4 and S5 for statistical analyses for (**D**) and (**E**).

After optimizing the treatment conditions, gene expression levels were compared between MOD(OMe)-000794 (treatment) and MOD-scrambled (control) in three biological replicates. ~483 Differentially expressed genes (DEGs) with a false discovery rate (FDR)-adjusted *p*-value < 0.05 are presented by heatmaps (Fig. S15A and Dataset S1). Further analyses highlighted DEGs related to ribosomal proteins (Fig. 5A), chaperones (Fig. 5B), and tRNAs (Fig. S15B) that were upregulated by MOD(OMe)-000794 (Table S1), suggesting that treatment with MOD(OMe)-000794 likely affected protein translation and folding. Conversely, genes associated with putative hemin uptake and purine metabolism pathways were significantly downregulated in the treatment group (Fig. S15C, D and Table S1). Furthermore, DEGs with at least 2-fold changes (*p* < 0.001) were presented by the volcano plot (Fig. 5C), and real time PCR (RT-PCR) was performed in three biological replicates to validate representative genes belonging to the same operons as predicted previously (Fig. 5D) [39]. To put these genes into a functional context, we performed a pathway enrichment analysis of differentially regulated genes based on the Kyoto Encyclopedia of Genes and Genomes (KEGG) database (Fig. S15E). The top five most differentially regulated pathway terms are ribosome, RNA degradation, purine metabolism, pentose phosphate pathway and fatty acid/lipid biosynthesis, which were affected by MOD(OMe)-000794 compared to MOD(OMe)-scrambled control.

The upregulation of chaperons and ribosomal proteins by MOD(OMe)-000794 relative to MOD(OMe)-scrambled appears to be counterintuitive since we previously showed that tsRNA-000794 resulted in a global translation attenuation in *Fn* ATCC 23726 using a click chemistry labeled amino acid [14]. However, it has been reported that the expression of certain ribosomal proteins increased when bacteria were challenged with ribosome-targeting antibiotics [40, 41]. For example, antibiotics targeting the 50 S subunit of the ribosome (chloramphenicol or its analog thiamphenicol) or disruption of ribosome assembly via overexpression of a translational repressor ribosomal protein can upregulate the levels of ribosomal protein mRNAs, tRNAs and rRNAs in *E. coli*, suggesting a negative feedback mechanism to cope with translation suppression [40, 41]. Indeed, when *Fn* ATCC 23726 were treated with thiamphenicol at a sub-minimal inhibitory concentration (1 μg ml^−1^), representative genes associated with hemin transport, chaperons, ribosomal protein mRNAs and tRNA clusters mirrored the changes found in MOD(OMe)-000794, with the exception that the putative purine biosynthesis genes showed the opposite changes in gene expression (Fig. 5E). Thus, we hypothesized that one of the mechanisms for tsRNA-mediated growth inhibition could be targeting ribosomes such that impaired protein translation leads to mis-folding of proteins and subsequent recruitment of protein chaperons. Since the sequence of MOD(OMe)-000794 matches part of the full tRNAs in *Fn* [14], it is plausible that MOD(OMe)-000794 acts as decoy tRNA to target the ribosome or rRNA and interfere with protein translation as reported previously [42]. To test the hypothesis, we biotinylated tsRNAs at either 5’ or 3’ and performed RNA affinity pulldown from the total cell lysate of *Fn* 23726. 50 S ribosomal proteins were found to be enriched in biotinylated MOD(OMe)-000794 or −020498 relative to that of biotinylated MOD(OMe)-scrambled (Dataset S3). Therefore, our data indicate that MOD(OMe)-000794 induced a potent translational attenuation in *Fn*, likely through targeting ribosome components and triggered upregulation of corresponding genes to ameliorate the stress.

**Fig. 6:**
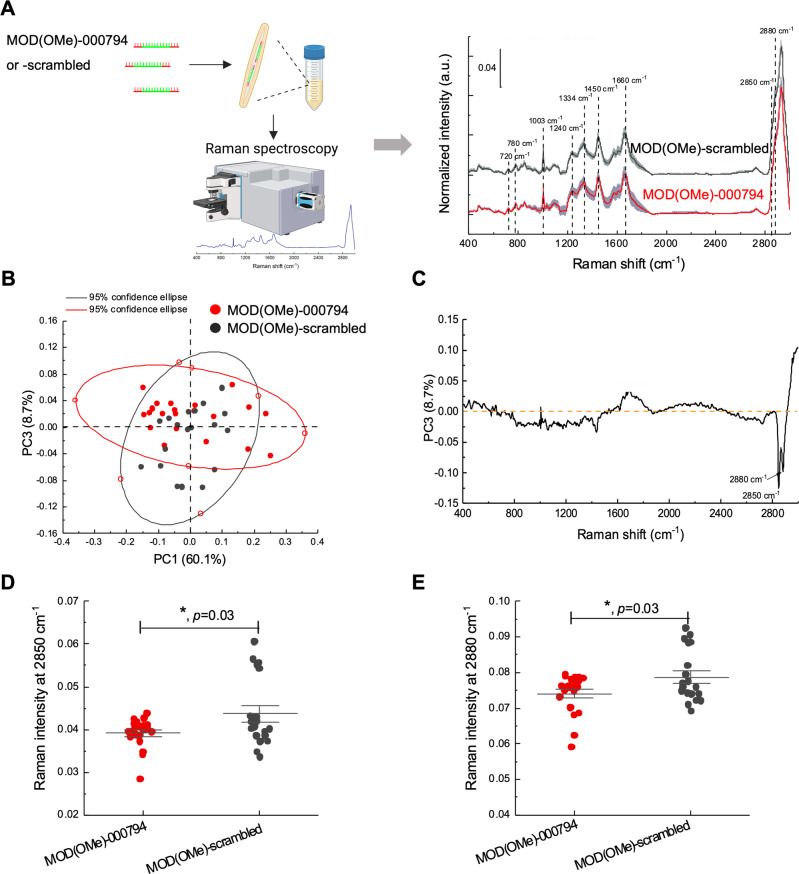
Raman spectroscopy analysis of *Fn* with MOD-tsRNA treatment. **A** Raman spectral signatures of *Fn* ATCC 23726 subject to MOD(OMe)-000794 versus MOD(OMe)-scrambled RNA control. The figure is created by BioRender. **B** Three-dimensional principal component analysis (PCA) of the Raman spectra showing PC3 contributes to the most difference between MOD(OMe)-000794 and MOD(OMe)-scrambled RNA control in *Fn* ATCC 23726. **C** Multiple Raman peaks reflecting chemicals such as proteins, especially proteins with aliphatic amino acids, glutamate, lipids were the major composition of PC3, suggesting differential *Fn* metabolic states when subjected to MOD(OMe)-000794 or MOD(OMe)-scrambled RNA treatment. Raman peak 2850 cm^−1^ (lipids and lipopolysaccharides) (**D**) and 2880 cm^−1^ (aliphatic amino acids) (**E**) are significantly reduced in MOD(OMe)-000794 compared to MOD(OMe)-scramble treatment control. **D** and **E** are means ± SEM of three independent experiments, and data were analyzed with the unpaired student t-test.

To further probe how MOD(OMe)-000794 impacted *Fn* ATCC 23726 at the molecular and cellular levels, we adopted Raman spectroscopy that has been shown to effectively measure different states of bacteria in a label-free manner. The Raman spectrum represents an ensemble of molecular vibrations, providing comprehensive but complex data reflecting the metabolism and chemical compositions of the cells exposed to various drugs of different concentrations or durations [43]. To characterize the phenotypic differences between MOD(OMe)-000794 and MOD(OMe)-scrambled RNA control treatments in *Fn* ATCC 23726, we collected the same batch of cells that were used for the aforementioned bacterial RNAseq. Formaldehyde-fixed bacteria were subject to Raman spectra acquisition to measure different metabolic states and cellular compositions. Several typical Raman peaks were identified corresponding to 720/780 cm^−1^ (DNA/RNA), 1003 cm^−1^ (phenylalanine), 1240 cm^−1^/1450 cm^−1^/1660 cm^−1^ (Amide III/II/I peaks), 2850 cm^−1^ (lipids and lipopolysaccharides), 2880 cm^−1^ (aliphatic amino acids) (Fig. 6A). Through three-dimensional principal component analysis (PCA) of the Raman spectra, a global difference was detected between MOD(OMe)-000794 and MOD(OMe)-scrambled RNA control treated *Fn* ATCC 23726 (Fig. S16A). To understand which principal component contributes to the highest difference, the three-dimensional PCA was projected into three two-dimensional PCA plots. It was shown that PC3 contributes to the most difference between MOD(OMe)-000794 and MOD(OMe)-scrambled RNA control in *Fn* ATCC 23726 (Fig. 6B, and Fig. S16B, C). To identify which components from the PC3 underlined the phenotypic differences in the MOD(OMe)-000794 treatment group, we extracted the spectral information from PC3 (Fig. 6C). Of note, peaks associated with the highest reduction from the MOD-tsRNA-00094 treatment are associated with proteins (containing aliphatic amino acids, 2880 cm^−1^) [44], lipids [44] and fatty acids (2850 cm^−1^) (Fig. 6D, E) [44]. The reduction of lipids and fatty acids data agreed with the KEGG analysis from RNAseq, where the fatty acid metabolism represented the top five most enriched terms with gene downregulation (Fig. S15E). Additionally, downregulation of proteins containing aliphatic amino acids is in line with the putative ribosome-inhibiting roles of MOD-tsRNAs as evidenced in the RNAseq (Fig. 5). Our data from Raman spectroscopy provided complementary evidence indicating the MOD-tsRNA-mediated interference of lipid metabolism and protein translation in *Fn*.

## Discussion

The chemical instability of sRNA molecules in general presents a formidable barrier to understanding the cross-kingdom functions of host sRNAs as well as developing RNA-based biologics for therapeutic applications. Over the last decades, chemically modified RNA nucleotides have greatly improved the nuclease resistance of RNAs while preserving their functionality. These efforts have propelled the development of nucleic acid-based genetic tools and therapeutics, including three recent FDA-approved small interfering RNA inhibitors for metabolic diseases, messenger RNA for vaccines, and CRISPR guide RNA for genome editing [21–24]. Building on the success of existing RNA technologies, we chemically modified host-derived tsRNAs as a new class of antimicrobial to target bacteria in a species- and sequence-dependent manner. Given the increasing implications of *Fn* in periodontal diseases, preterm birth, and cancer development and chemoresistance [18–20], it can be highly desirable to further enhance and characterize the anti-*Fn* activities of new MOD-tsRNA variants through different chemical modifications for the following reasons. First, we have found that certain *Fn* clinical isolates and type strains (e.g., *Fn* 25586) were less susceptible to inhibition by MOD(OMe)-000794 than *Fn* 23726, which likely reflects differences at the bacterial subspecies level as well as strain variation [29]. Further investigation can be conducted to investigate the mechanisms underlying the observed difference in tsRNA sensitivity. Second, bacteria are generally more resistant to growth inhibition in biofilms than in planktonic states, the former of which is represented in dental plaques [45]. Third, interactions between different bacteria have been shown to confer resistance to certain inhibitors [46]. Therefore, the current modifications explored in this study set the stage for future efforts to harness host-derived tsRNAs towards target-specific antimicrobials in the native context such as polymicrobial biofilms. In terms of potential effects of MOD-tsRNAs on host cells, while we used a non-transformed, spontaneously immortalized normal oral keratinocyte cell line (NOKSI) in the study, for future translational investigation it is desirable to characterize the toxicity using primary human cells.

To dissect the mechanisms of inhibition, we performed bacterial RNAseq to profile gene expression changes in *Fn* comparing MOD(OMe)-000794 to the scrambled control RNA. Most upregulated genes are associated with ribosomal proteins, tRNAs and protein chaperones, which were further validated by quantitative RT-PCR. Of note, the KEGG analysis indicated that the protein translation represents the most enriched pathways targeted by MOD(OMe)-000794. The seemingly paradoxical upregulation of ribosome proteins during growth inhibition has been well documented in the literature and represents a hallmark of bacterial response when subjected to ribosome-targeting antibiotic treatments [40, 41, 47, 48]. In addition to their well-known inhibition of translation by interfering ribosomal functions, many of the ribosome-targeting antibiotics can directly bind to 30 S or 50 S ribosomal subunit precursors and inhibit the ribosomal assembly. These will often lead to increased expression of ribosomal protein-encoding genes [41, 47]. We speculated that MOD-tsRNAs may function as a new class of ribosome-targeting antimicrobials as the same set of genes were also upregulated when *Fn* was treated with a ribosome-targeting antibiotic, thiamphenicol. The upregulation of ribosomal protein mRNAs, tRNAs and protein chaperons may reflect bacterial stress responses during global translation inhibition mediated by MOD(OMe)-000794. To further corroborate the hypothesis, we carried out a biotinylated tsRNA pulldown assay and found that MOD(OMe)-000794 indeed recognizes several ribosomal proteins compared to MOD-scrambled RNA control. In addition to targeting the translation machinery, putative genes related to purine synthesis and hemin uptake are key downregulated genes and pathways upon the challenge of MOD(OMe)-000794. Since purine and hemin are essential for bacterial DNA synthesis and anaerobic growth, it is plausible to speculate that MOD-tsRNAs directly or indirectly interfere with multiple cellular functions to inhibit the growth of *Fn*.

The present work has also led to many intriguing questions. First, while findings from the global RNAseq implicate translation-related genes and pathways in the growth inhibition of *Fn*, the specific targets of MOD-tsRNAs remain to be determined. It is possible that MOD-tsRNAs may directly interfere with ribosomal proteins to attenuate global mRNA translation in *Fn* mainly for two reasons: (1) No direct sequence complementarity was detected between the two *Fn*-targeting tsRNAs and *Fn* ATCC 23726 RNAs, thus arguing against an antisense mechanism. However, the central 21 nucleotides of tsRNA-000794 and 020498 match with the sense sequences of *Fn* tRNAs, suggesting that tsRNAs may be mis-incorporated into the ribosome machinery during active translation as the “decoys” of tRNAs. (2) Despite current understanding of tsRNAs originated from studies in eukaryotic cells, most, if not all, tsRNAs have been shown to directly associate with RNA-binding proteins to affect mRNA stability and translation. Indeed, our biotinylated tsRNA pulldown assay supports the recognition of ribosomal proteins by tsRNAs. Second, it remains unclear how the tsRNAs define the species specificity. While our current findings suggest that the specificity is at least in part determined by an active uptake mechanism for tsRNAs in *Fn*, a putative transporter machinery for host sRNAs is yet to be identified in *Fn*. Of note, efforts have been made towards the identification of putative RNA importer proteins that facilitate internalization of extracellular sRNAs for intercellular or cross-kingdom gene modulation, such as Systemic RNA Interference Deficiency-1 (SID-1) in *C. elegan*, and the nematode homolog protein SID-1 transmembrane family member 1 (SIDT1) in mammalian cells. To the best of our knowledge, however, no such importer protein has been found in bacteria. Lastly, in addition to the selective uptake of certain tsRNAs by different bacteria, it is also possible that the intracellular targets of certain tsRNAs are only present in some bacteria such as *Fn*, which can dictate the bacterial range of different host-derived sRNAs in the context of cross-kingdom interactions. While we have focused on two tsRNAs in this present work, future work can investigate the full spectrum of host-derived tsRNAs or other sRNA molecules to uncover the fundamental mechanisms and full potential of host sRNAs underlying the host-microbiota interactions.

In summary, our work highlights an opportunity to use chemically modified RNA nucleotides to understand and harness host-derived tsRNAs to target pathobionts.

## Supplementary information

Supplemental Information

Supplemental Dataset S1

Supplemental Dataset S2

Supplemental Dataset S3

## Data Availability

All data are available in the main text and the supplementary materials.
