# Supplementary material for: Targeting Fusobacterium nucleatum through chemical modifications of host-derived transfer RNA fragments
Source: ISME J. 2023 Apr 1;17(6):880–90. doi: 10.1038/s41396-023-01398-w (PMC10202947; doi:10.1038/s41396-023-01398-w)
Supplement: Supplementary file 1 — Supplemental Information [file 41396_2023_1398_MOESM1_ESM.docx]

**SI Materials and Methods:**

*Chemicals*

All chemicals and cell culture broth were purchased from Fisher Scientific International Inc. (Cambridge, MA, USA) unless otherwise noted, and were of the highest purity or analytical grade commercially available. DNA and RNA oligos were ordered from MilliporeSigma (St. Louis, MO, USA) and Integrated DNA Technologies ([Coralville, IA](https://www.google.com/search?sxsrf=ALeKk01lQ3tdmviLYKFhcTMeUP-b7OTfgQ:1616435039456&q=Coralville,+Iowa&stick=H4sIAAAAAAAAAOPgE-LSz9U3MMnKyLVIUuIAsUsMDDK0tLKTrfTzi9IT8zKrEksy8_NQOFYZqYkphaWJRSWpRcWLWAWc84sSc8oyc3JSdRQ888sTd7AyAgBIlqg5WgAAAA&sa=X&ved=2ahUKEwjf1NDiucTvAhXqRt8KHfI2AhQQmxMoATAgegQIJxAD), USA). All molecular cloning reagents including restriction enzymes, competent cells, and the Gibson assembly kit were purchased from New England Biolabs ([Ipswich, MA](https://www.google.com/search?sxsrf=ALeKk03fLfjFfarea2TmknvyUFtpTUm2IA:1616436007883&q=Ipswich+,+Massachusetts&stick=H4sIAAAAAAAAAOPgE-LSz9U3MDKvyMoyV-IEsQ1zzQsqtbSyk63084vSE_MyqxJLMvPzUDhWGamJKYWliUUlqUXFi1jFPQuKyzOTMxR0FHwTi4sTkzNKi1NLSop3sDICAB6fQlNiAAAA&sa=X&ved=2ahUKEwj98LSwvcTvAhXGmOAKHakdAMUQmxMoATAcegQINhAD), USA) .

*Bacterial Strains and Growth Conditions*

*Fn* ATCC 23726, 25586, *Sm* ATCC 6249, and *Pg* ATCC 33277 were purchased from the American Type Culture Collection (Manassas, VA, USA). *Fn* clinical tumor isolates were generous gifts of Dr. Garrett Wendy at the Harvard T.H. Chan School of Public Health. Bacterial glycerol stocks are routinely verified by 16S sequencing. *Fn* strains and *Pg* were cultured in liquid Columbia broth (CB) or on CB agar plates containing 5% defibrinated sheep blood (Hemostat laboratories, Dixon, CA, USA), and incubated at 37°C in an anaerobic chamber (Sheldon Manufacturing, Cornelius, OR, USA) containing 5% H2, 10% CO2, 85% N2. *Sm* were cultured in Brain-Heart Infusion (BHI) broth. *E. coli* K-12 were cultured in lysogeny broth (LB) media and incubated at 37 °C under aerobic conditions.

*tsRNA Stability Test in Saliva*

Saliva samples were collected from human subjects under under the Forsyth IRB protocol number 21-02. Samples were filter sterilized to obtain cell-free saliva and kept at -80°C until use. A mixture of three RNAs (two MOD-tsRNAs and one MOD-scrambled) or three corresponding naturally occurring ones were added to fresh collected saliva at a working concentration of 10 nM per each RNA under the Forsyth Institute IRB protocol number 21-02. 100 μl sample was frozen immediately by liquid nitrogen as the start point (0 h) and stored at -80 °C for the next step. Samples were incubated at 37 °C for 15 min, 30 min, 1h before snap freezing. The collected samples were cleaned up by the small RNA collection protocol using Zymo RNA Clean & Concentrator^TM^-25 according to the instructions. tsRNAs were reverse transcribed to cDNA with a HiFiScript cDNA Synthesis Kit (CoWin BioSciences, Cambridge, MA, USA) by stem-loop primers. The detailed primer sequences are provided in Tables S2 and S3. cDNA was amplified and quantified by a QuantStudio 3 Real-Time PCR System (ThermoFisher, Waltham, MA, USA).

*MTT Cell Proliferation Assay*

The normal oral keratinocyte (NOKSI) cell line was a gift from Dr. Silvio Gutkind and tested negative for mycoplasma contamination. Cells were cultured with a defined keratinocyte serum-free medium. The in vitro cell toxicity test was performed using the MTT assay (MilliporeSigma). MTT stock solution was prepared in sterilized 1xPBS and used with 1:10 dilution. NOSKI cells were seeded in 96 well plates at a concentration of 5000 cells/well and incubated with 100 µL of MTT reagent for 3 h inside the cell incubator. The converted dye is solubilized with dimethyl sulfoxide at the end of the incubation period. Finally, the cell viability in each well was measured at a wavelength of 570 nm with background subtraction at 650 nm.

*Sodium Azide Treatment*

Sodium azide was prepared at a stock concentration of 1 M in water. *Fn* ATCC 23726 with a starting OD_600_ of 0.1 were mixed with sodium azide at a concentration range of 0 to 10 mM. Moreover, tsRNA-Cy3 was added to the above scenarios at a working concentration of 500 nM. Samples were then incubated at anaerobic chamber overnight before taking out for image acquisition and OD_600_ measurement.

*Fluorescence Microscopy of tsRNA-Alexa 488 Labeled Fn Strains*

3' Alex488 labeled tsRNA were reconstituted in 1xPBS. Overnight-grown *Fn* was diluted to an OD_600_ of 0.1 and treated with 250 nM tsRNA-000794-Alexa 488, tsRNA-020498-Alexa 488, and scrambled RNA-Alexa 488 control for 24h at an anaerobic chamber followed by three washes with 0.9% NaCl. Washed samples were then sandwiched between a cover glass and a SUPERFROST® PLUS-Adhesion Slide (Electron Microscopy Sciences, Hatfield, PA, USA). Samples were then immediately imaged by a Nikon, Epi-fluorescence microscope.

*SYTOX Green Assay*

*Fn* ATCC 23726 after treatment with MOD(OMe)-000794, MOD(OMe)-020498, and MOD(OMe)-scrambled RNA control was centrifuged at a speed of 17,000 × *g* for 5 min, then washed with 1×PBS. SYTOX green (ThermoFisher, catalog# S7020) was added to *Fn* at a working concentration of 5 μM in 1×PBS. Fluorescence intensity was then acquired after incubation at room temperature for 30 min. SYTOX green was excited at a wavelength of 488 nm and collected through a bandpass filter from 500-550 nm. An oil-immersion 63× (NA=1.4) objective (Zeiss Objective Plan-Apochromat) was utilized to image samples. Transmission images were acquired through a differential interference contrast setting. All the images were acquired under the same image acquisition setting and analyzed by FiJi and R. To quantify the fluorescence intensity from MOD(OMe)-000794 and MOD(OMe)-scrambled RNA control-treated *Fn*, integrated fluorescence intensity from the whole image was normalized by the total area of *Fn*. The higher the value indicates lower viability.

*RNA Isolation*

Overnight bacteria culture was diluted to OD_600_ of 0.2 before the tsRNA treatment. 10 µL 100 µM MOD(OMe)-000794, MOD(OMe)-scrambled RNA, and 1xPBS were added to 2 ml diluted bacterial culture separately with three biological repeats at the 500 nM working concentration. Cell pellets were collected at 5 h at 13,000 x *g* 2min followed by snapping freeze in liquid nitrogen and stored at -80 °C until RNA extraction. RNA was extracted by RNApure Tissue & Cell Kit (CoWin Biosciences, catalog# CW0560S) as manufacturer’s instructions except for DNase I treatment. RNA was eluted with 30 µL 1x TE buffer (10 mM Tris-HCl, 0.1 mM EDTA, pH 7.5) twice followed by TURBO DNA-free kit (ThermoFisher, catalog# AM1907) treatment at 37 °C for 20 min. 5 µL of Dnase-treated RNA was used for reverse transcription later and the remainder RNA was stored at -80°C until RNA Sequencing. For thiamphenicol treatment group, overnight bacterial culture was diluted to OD_600_ of 0.5 followed by adding 6uL 1 mg mL^-1^ thiamphenicol or vehicle control (EtOH) to 6 mL diluted bacterial culture. After 5h treatment, collected the pellets and isolated RNA as description.

*Real-Time Polymerase Chain Reaction (RT-PCR)*

Primers were designed by NCBI Primer BLAST and synthesized by MiliporeSigma. cDNA was synthesized from total RNA by HiFiScript cDNA Synthesis Kit (CoWin Biosciences). RT-PCR was performed in 96-well plates format using FastSYBR Low Rox (CoWin Biosciences) on Quantstudio 3 Real-Time PCR System (ThermoFisher) using the following protocol: 95 °C for 20 s, 40 cycles of 95 °C for 3 s, and 60°C for 30 s, and a final cycle of 95°C for 15 s, 60 °C for 1 min, and 95 °C for 15 s. Relative gene expression was calculated by 2^–∆∆Ct^ method using 16S RNA as the reference gene. RT-PCR was performed with three technical repeats and three biological repeats. Primers used for RT-PCR are shown in Tables S2 and S3.

*Biotinylated tsRNA Affinity Pulldown from Bacterial Lysate and Mass Spectrometry*

Dynabeads® M-270 Streptavidin beads (ThermoFisher, catalog# 65305) and streptavidin agarose resin (G-Biosciences. St. Louis, MO. U.S.A) were washed according to the instructions and blocked by 1 mg mL^-1^ BSA at RT for 1 h. M-270 beads and resin were washed by 3xTBS (high salt, 150 mM Tris HCl, 0.45 M NaCl, pH 7.4) and 1xTBS (medium salt, 50 mM Tris HCl, 0.15 M NaCl, pH 7.4) three times respectively and resuspended in the same volume buffer as the initial volume of beads taken from vial by 3xTBS and 1xTBS. 50 mL bacterial culture was harvest by spinning at 4000 rpm 15min followed by 3 times 1xPBS washing steps. Cell pellet was rotated at RT for 20 min in 3 mL cell lysis buffer (50 mM Tris HCl, 0.15 M NaCl, pH 7.4, 1 mM DTT, 0.5% (v/v) NP-40, 50 μg mL^-1^ lysozyme and protease inhibitor cocktail EDTA free). Lysates were sonicated on ice with 5 s “on” and 5 s “off” for a total 5 min at 50% power (Soniprobe, Dawe Instruments, England) for 3-4 times until the lysate was transparent and cleared by centrifugation. Heparin was added to the clear extracts with 100 μg mL-1 working concentration followed by incubation with precoated resin at 4 °C for 30 min and quantified for protein concentration by DC Protein Assay reagent package. Before incubating with pretreated M-270 beads, 1 mM MgCl_2_, 1 mM ATP and 40U mL^-1^ RnaseOUT (ThermoFisher, catalog# 10777019) were added to the precleared cell lysates. 5’ or 3’ Biotin-tsRNA with the final concentrations 500 nM was added to 10 μL precoated M-270 beads and incubated on ice for 30mins. After 3 times of washing by 1xTBS, the precleared lysates were aliquoted to the pretreated M-270 beads equally and incubated at RT for 2 h on the rotator. M-270 beads were washed for 10 min at RT with wash buffer (50 mM Tris HCl, 0.15 M NaCl, pH 7.4, 0.5%(v/v) NP-40) twice followed by 1xTBST (25 mM Tris HCl, 0.15 M NaCl, pH 7.4, 0.05% (v/v) Tween^TM^-20) twice. Beads were sent to Poochon Scientific, CT, USA for mass spectrometry analysis.

*Raman Spectroscopy*

Cells were dissociated into single cells and fixed in 4% (v/v) paraformaldehyde for 15 min and were washed three times with 1xPBS. Before Raman measurements, the fixed cells were dropped onto an aluminum-coated Raman substrate to be air dried. Raman spectra were acquired using an HR Evolution confocal Raman microscope (Horiba Jobin-Yvon) equipped with a 532-nm neodymium-yttrium aluminum garnet laser. The laser power on cells was 12 mW after attenuation by neutral density filters. An objective with a magnification of 100× was used to focus single cells with a laser spot size of ∼1 μm^2^, and Raman scattering was detected by a charge-coupled device cooled at −70 °C. The spectra were acquired in the range of 400 cm^−1^ to 3,000 cm^−1^ with a 600 grooves per mm diffraction grating. A mapping mode was used to characterize single cells pooled from three biological replicates, and the acquisition parameters were 20 s per spectrum by averaging two times, around 20 spectra from three biological replicates. Each sample was performed in three biological replicates and three technical replicates. All SCRS were preprocessed by cosmic ray correction and polyline baseline fitting with LabSpec 6 (Horiba Scientific). Spectral normalization was done by vector normalization of the entire spectral region (normalized by the norm). The choice of vector normalization was made to correct general instrumentation fluctuation as well as sample and experimental variables (e.g., thickness of the sample) without strongly interfering with the nature of the biological content. Normalization using a particular component, such as nucleic acids or Amide I peak, was not used here, to avoid any presumptions of specific biomolecular changes. Final data were presented through R and OriginPro.

**SI Figures:**


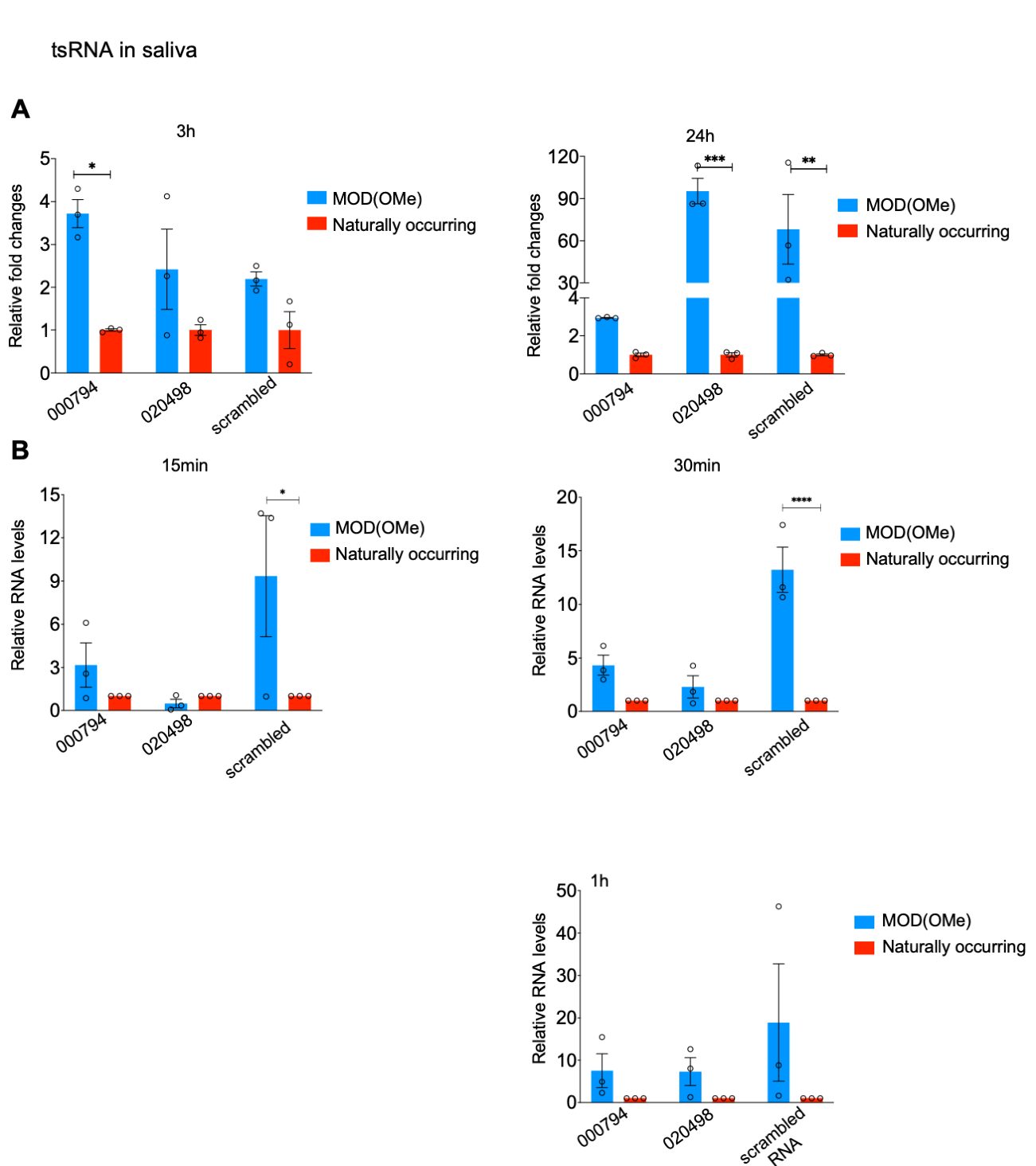


**Figure S1**: (*A*) The enhanced stability of MOD-tsRNA over naturally occurring tsRNA at 3 and 24 h timepoints in Columbia Broth. *n* = 3 technical replicates, *N* = 2 independent experiments. (*B*) MOD-tsRNAs are more stable than naturally occurring ones in saliva. *n* = 3 technical replicates, *N* = 3 independent experiments. The levels of intact MOD(OMe)-000794, MOD(OMe)-020498, MOD(OMe)-scrambled RNA, and corresponding mimics of naturally occurring RNAs were measured by the stem-loop reverse transcription PCR assay after incubation in the Columbia broth or saliva. Fold changes were normalized to the levels of naturally occurring tsRNAs at corresponded timepoints, which are shown as “1” on the y axis. Data were analyzed by the two-way ANOVA followed by Dunnett’s Bonferroni multiple comparison tests. **p* < 0.05, ***p* < 0.01, ****p* < 0.001, *****p* < 0.0001. Data are means ± SEM.


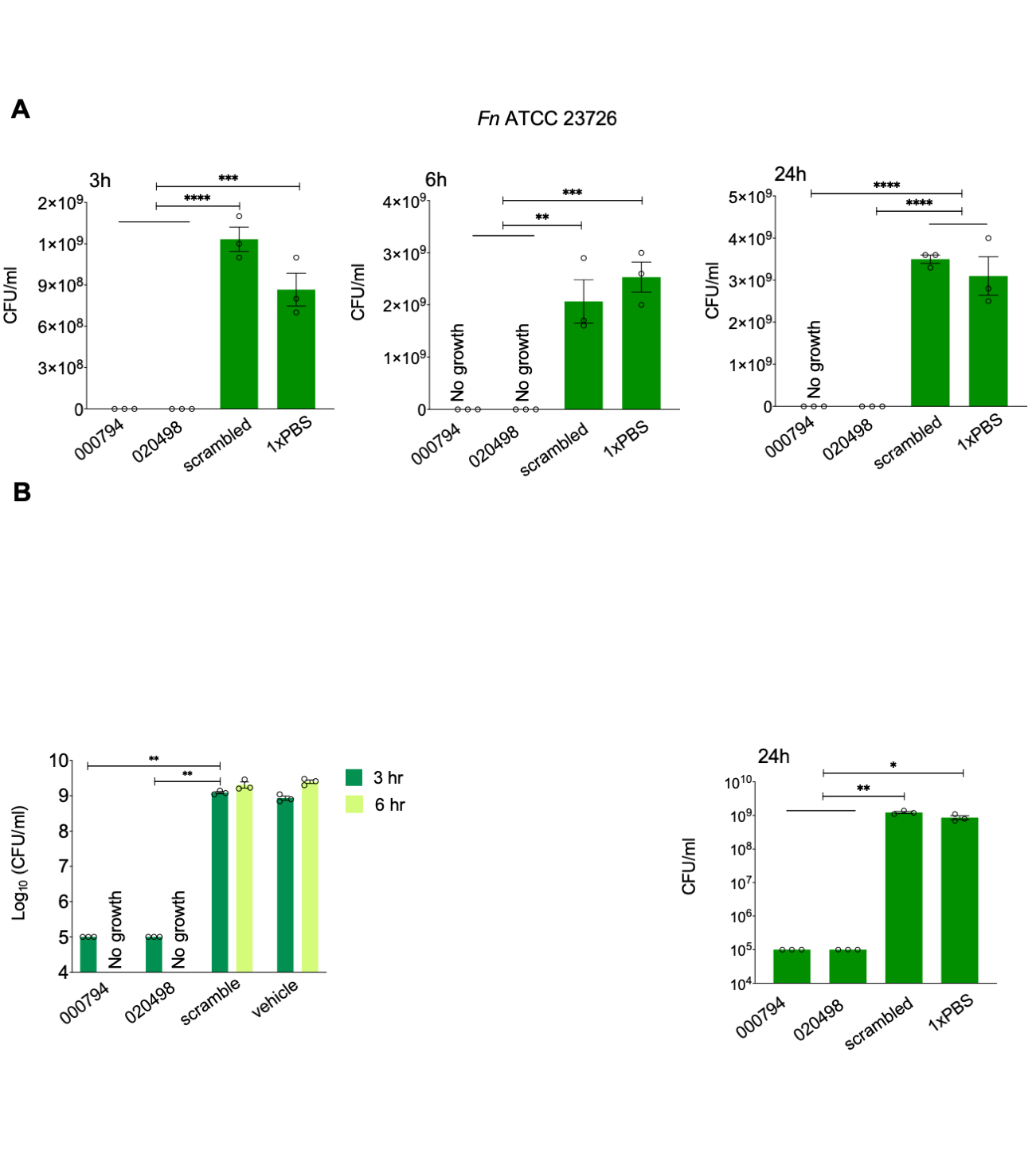


| **Day 2** | **Day 5** |
| --- | --- |
| 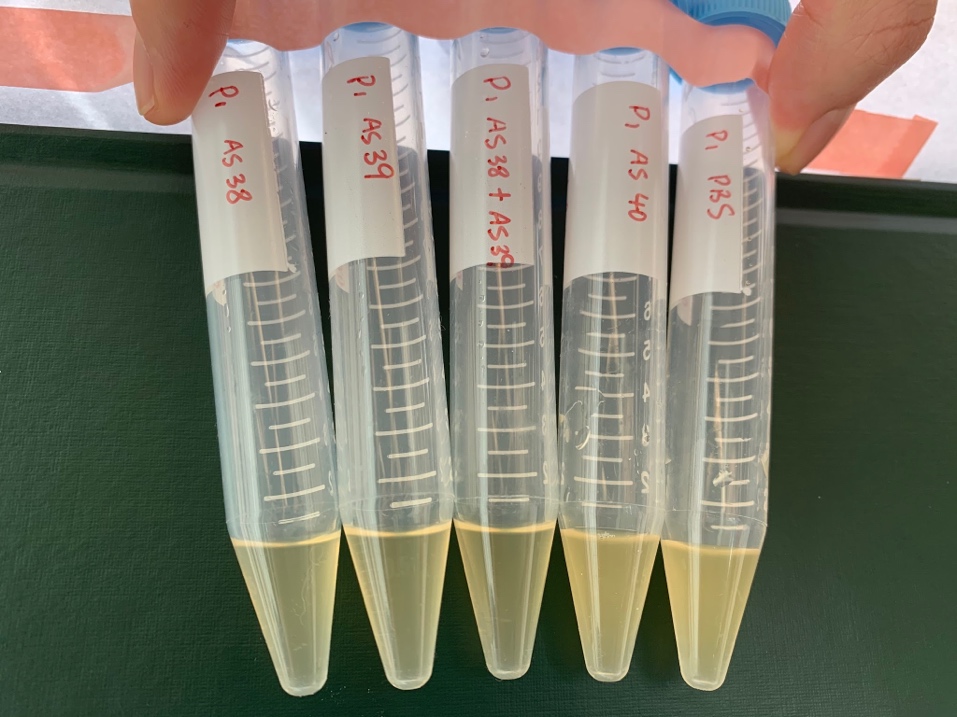 | 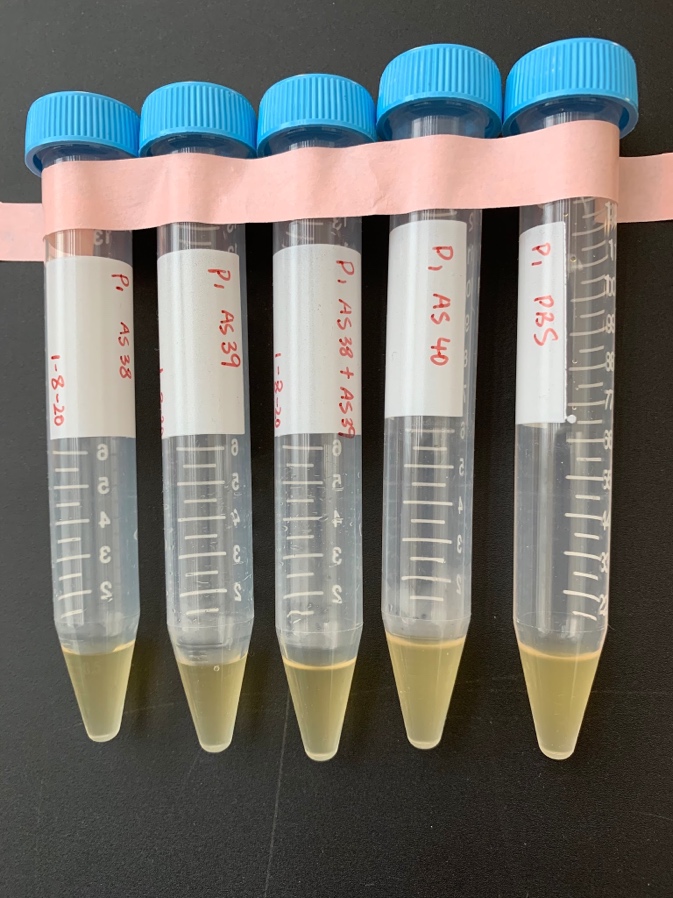 |

**Figure S2:** (*A*) Quantification of *Fn* ATCC 23726 CFUs after 3, 6 or 24 h treatment with 512 nM MOD(OMe)-000794, MOD(OMe)-020498, MOD(OMe)-scrambled and 1xPBS (vehicle control). Data were analyzed by the one-way ANOVA followed by Dunnett’s Bonferroni multiple comparison tests. ***p* < 0.0021, ****p* < 0.0002, *****p* < 0.0001. Data (means ± SEM) were representative of two independent experiments with three technical repeats. (*B*) *Fn* 23726 was treated with 500 nM MOD(OMe)-000794 (AS38), MOD(OMe)-020498 (AS39), AS38+AS39, MOD(OMe)-scrambled (AS40) or PBS for up to 24 h, and then replaced with fresh Columbia broth without any tsRNAs. Over the course of two and five days, only the scramble control and PBS treatment groups exhibited bacterial re-growth. Note that the 15 ml conical tubes were made of polypropylene and hence appeared translucent. Images are representative of two biological repeats.


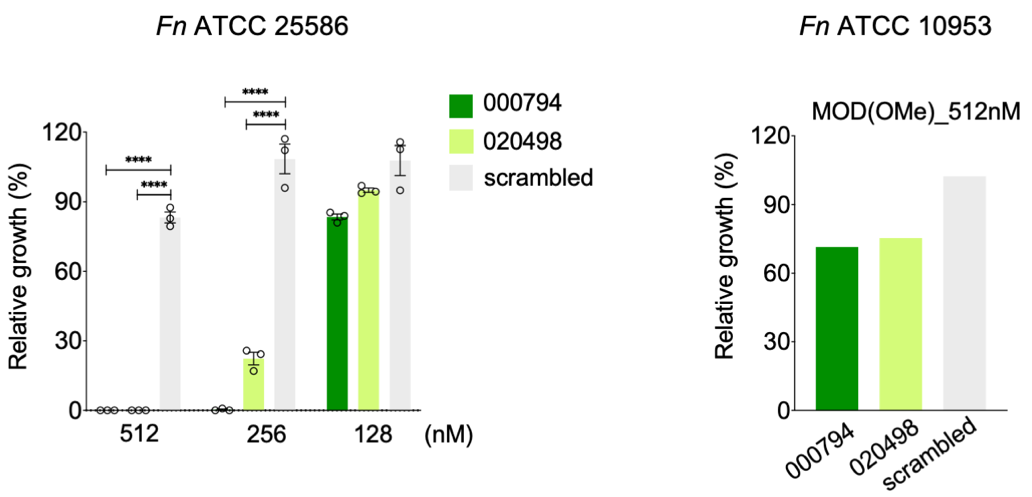


**Figure S3**: MOD(OMe)-000794 and MOD(OMe)-020498, but not MOD(OMe)-scrambled RNA, inhibited the growth of *Fn* ATCC 25586. Higher concentrations of MOD(OMe)-tsRNAs were needed than those in *Fn* ATCC 23726 as shown in Fig 1*C*. Data are analyzed by the two-way ANOVA followed by Dunnett’s Bonferroni multiple comparison tests. *****p* < 0.0001. Data are representative of two biological repeats with three technical repeats for *Fn* ATCC 25586.


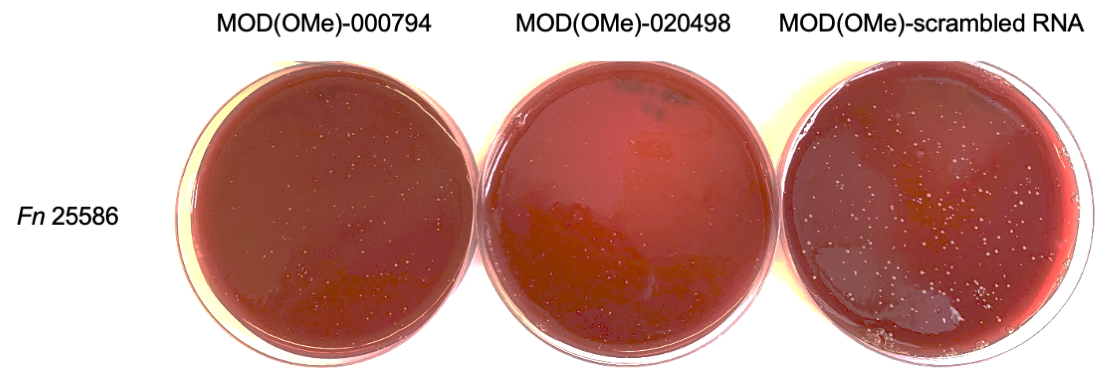


**Figure S4:** Representative images of plating *Fn* ATCC 25586 on nonselective blood agar after overnight treatment with 512 nM MOD(OMe)-tsRNAs in liquid culture under anaerobic conditions. Results are representative of two biological replicates.


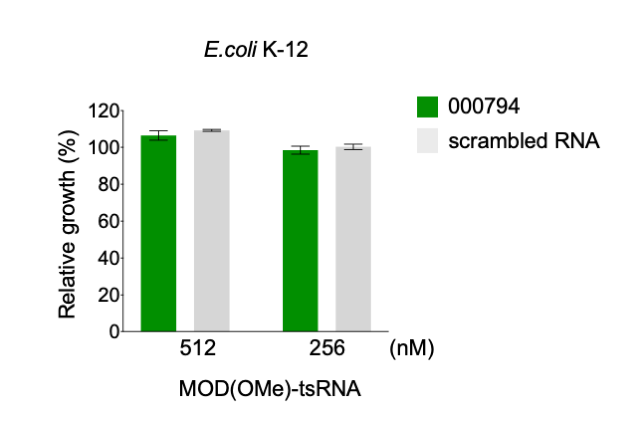


**Figure S5:** Lack of growth inhibition in *E. coli* K-12 after 24 h treatment with 512 or 256 nM MOD(OMe)-000794 and MOD(OMe)-scrambled.


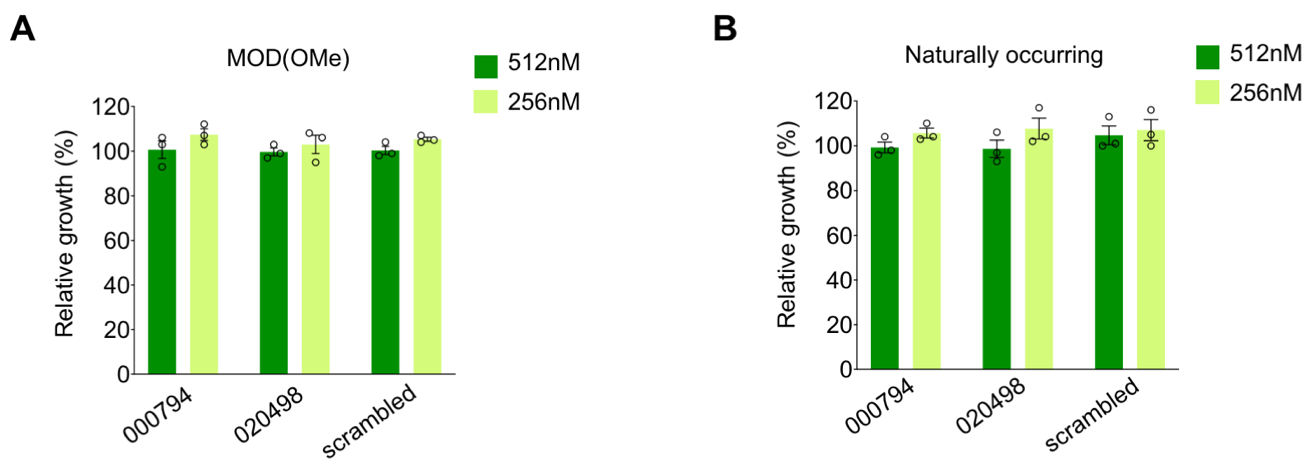


**Figure S6:** Relative growth rates of Normal Oral Keratinocytes-Spontaneously Immortalized (NOKSI) cells treated with MOD(OMe)-tsRNAs (*A*) or naturally occurring ones (*B*) for 48 h. At 48 h, cell proliferation was measured by the MTT assay, and the growth rates were normalized to the untreated control groups. Growth data = means ± SEM (n=3), and are representative of two biological replicates.


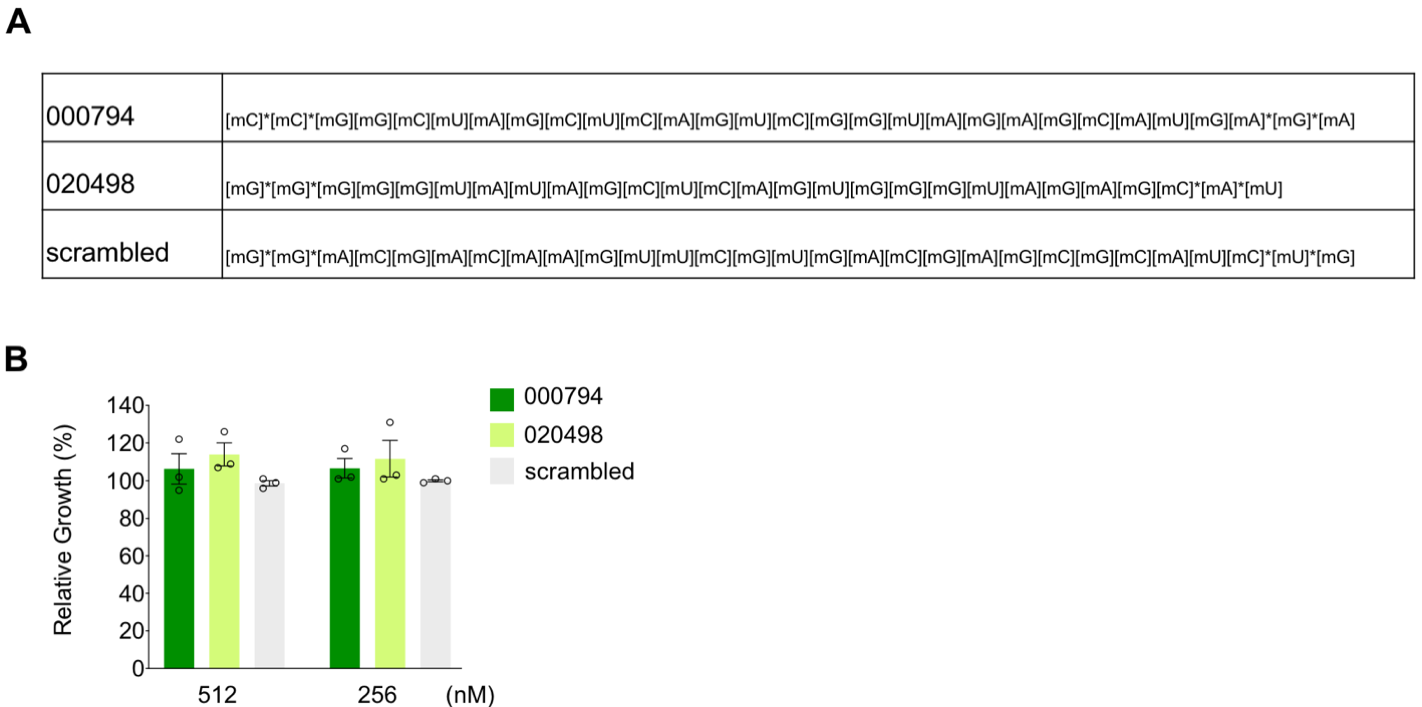


**Figure S7:** (*A*) Compositions and sequences of fully modified tsRNAs. * indicates a phosphorothioate (PS) bond, and [mA], [mU], [mC] and [mG] denote ribonucleotides with 2'-O-methylation. (*B*) Relative growth rates of *Fn* ATCC 23726 treated with fully modified tsRNAs. Full modifications of RNA backbone completely abolished the efficacy compared to partially modified tsRNAs (*i.e.*, MOD-tsRNAs shown in Fig. 2). Growth data = means ± SEM (n=3) and are representative of three biological replicates.

**
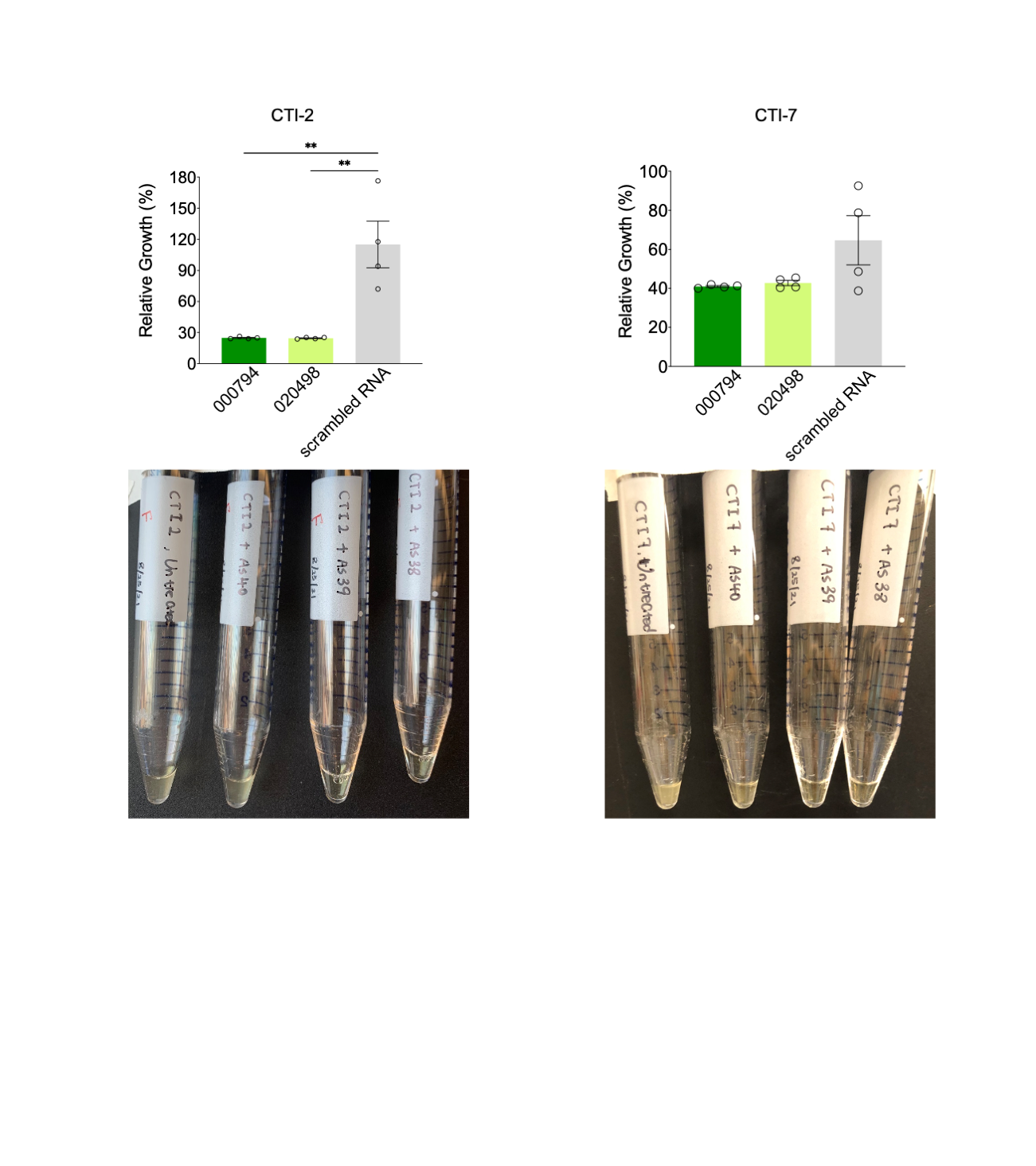
**

**Figure S8:** Growth inhibition of CTI-2 and CTI-7 by MOD(OMe)-000794 and MOD(OMe)-020498 relative to MOD(OMe)-scrambled RNA. Growth measurements were performed by OD absorbance, which are consistent with SYTOX Green fluorescence staining in **Figure 3**. AS38: MOD-tsRNA000794; AS39: MOD-tsRNA-020498; AS40: MOD-scrambled RNA. Data were analyzed by the unpaired t-test, **p ≤ 0.01.


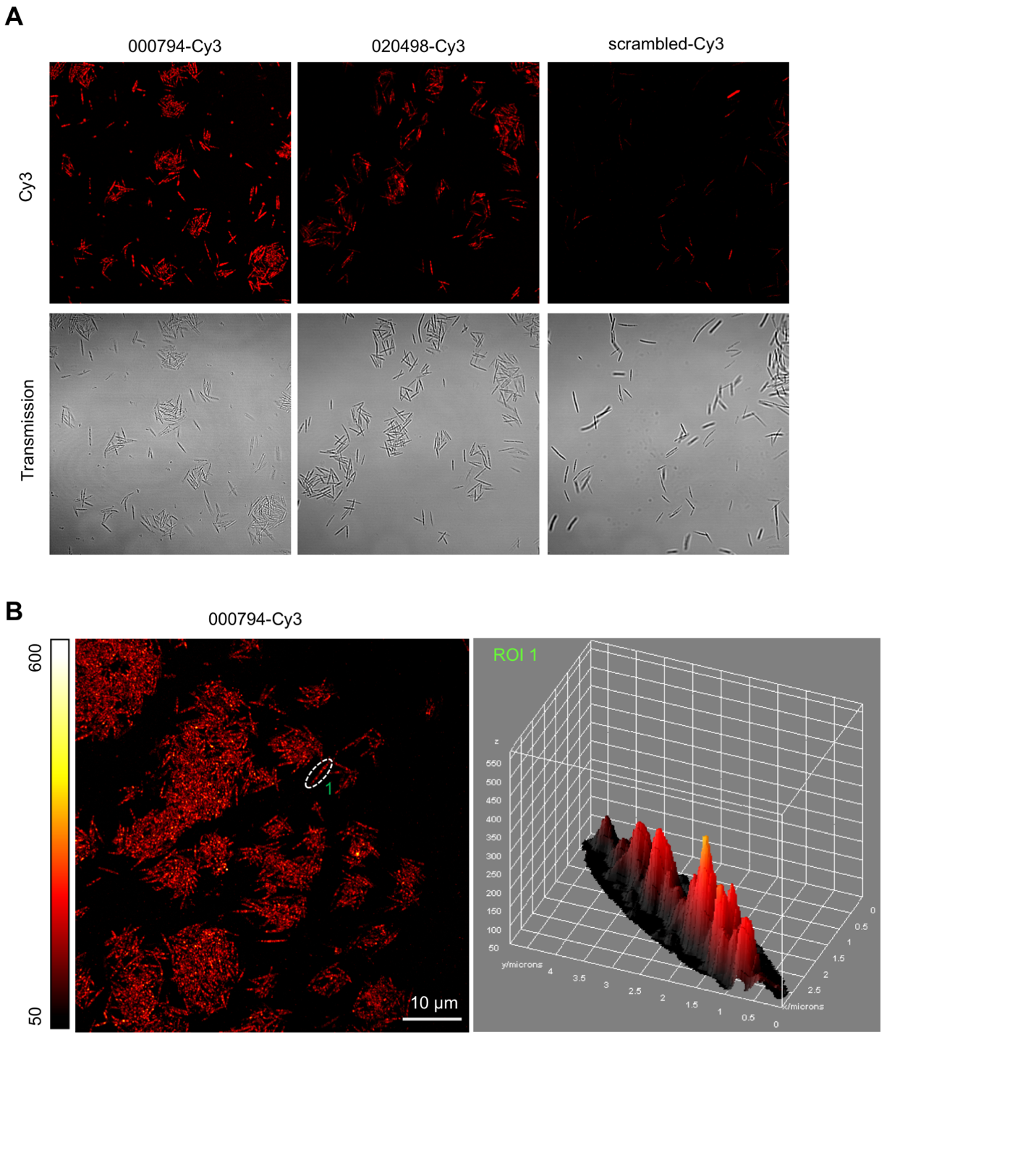


**Figure S9:** (*A*) Treatment of *Fn* ATCC 25586 with 128 nM tsRNA-000794-Cy3, tsRNA-020498-Cy3 or scrambled RNA-Cy3, and subsequent visualization by fluorescence microscopy. (*B*) Internalization of tsRNA-000794-Cy3 by *Fn* ATCC 25586 imaged by Airyscan confocal microscopy. Right: a randomly picked bacterium was analyzed with the height (z-axis) indicating the levels of tsRNA-000794-Cy3.


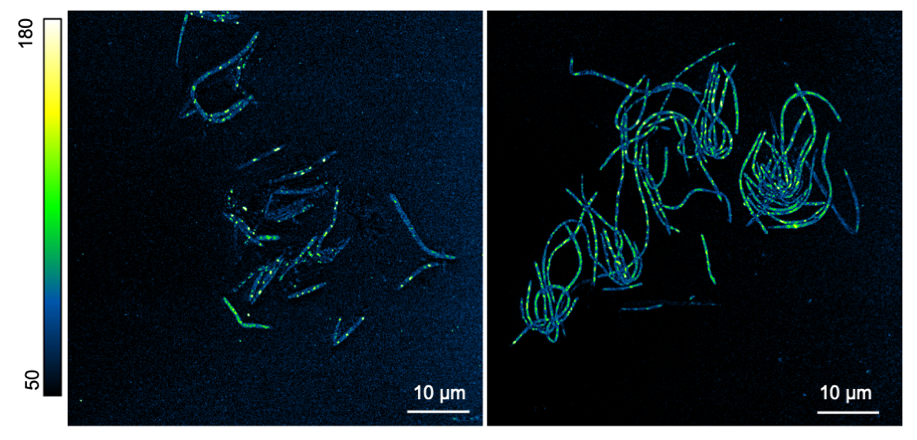


**Figure S10:** The uptake of tsRNA-000794-Cy3 by colon cancer-associated *Fn* isolates as imaged by Airyscan confocal microscopy.

**
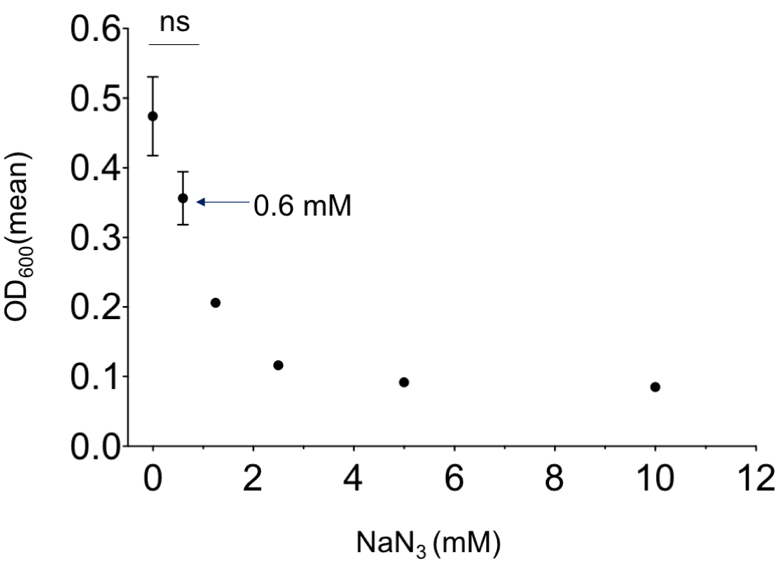
**

**Figure S11:** Effect of different sodium azide concentrations on the growth of *Fn* ATCC 23726. The growth of *Fn* was minimally reduced when treated by 0.6 mM sodium azide. Bacterial culture at the log phase were diluted to OD600 of 0.1, treated with NaN_3_ at a series of indicated concentrations, and subject to OD_600_ measurement after overnight incubation under anaerobic conditions.


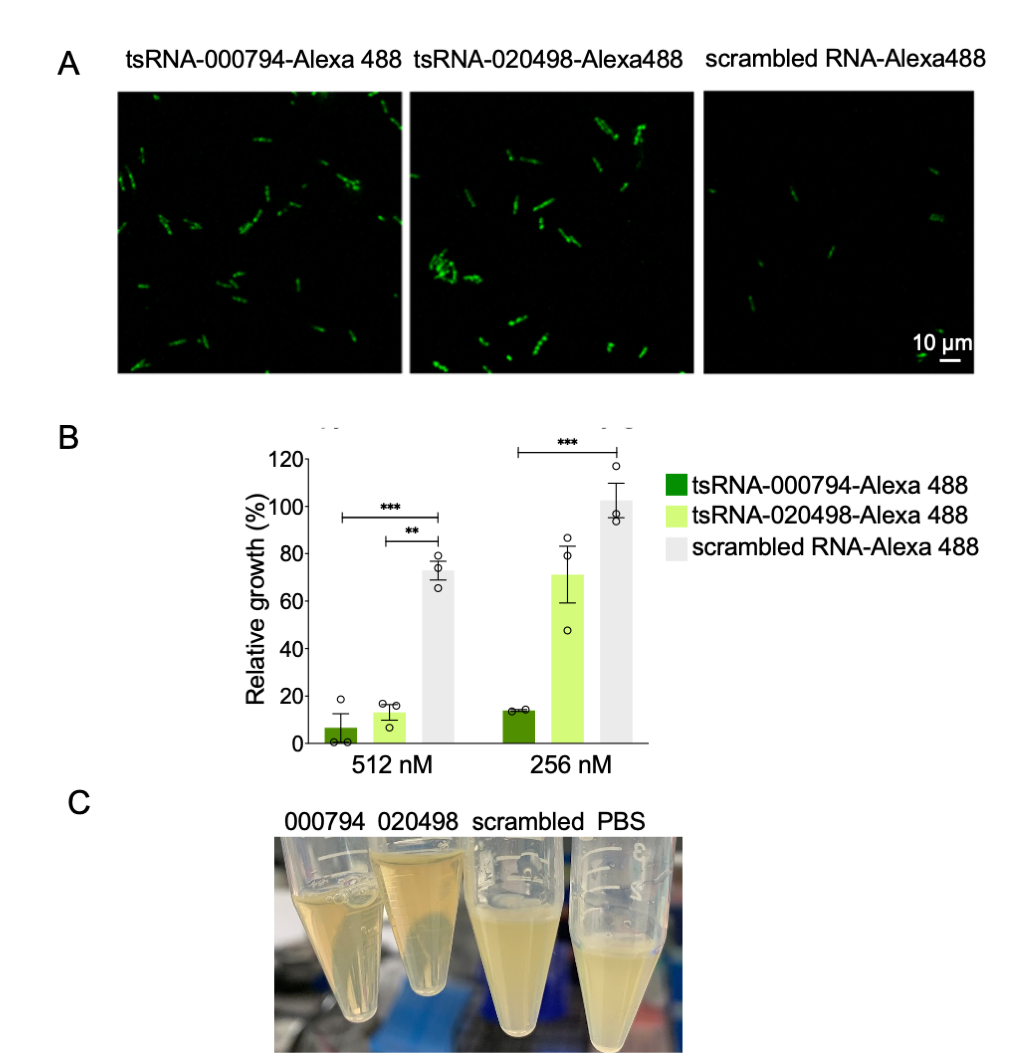


**Figure S12:** (*A*) Internalization of 256 nM Alexa 488-labeled tsRNA-000794, tsRNA-020498 and scrambled RNA control in *Fn* ATCC 23726. Confocal microscopy demonstrated enhanced uptake of tsRNA-000794-Alexa 488 and tsRNA-020498-Alexa488 relative to the scrambled control-Alexa 488. (*B*) Growth inhibition of *Fn* ATCC 23726 by Alexa-488-conjugated tsRNA-000794 and tsRNA-020498 but not the scrambled control. Data are analyzed by the two-way ANOVA followed by Dunnett’s Bonferroni multiple comparison tests. ***p* < 0.01, ****p* < 0.001. (*C*) Representative images of *Fn* ATCC 23726 after overnight treatment with 512 nM of Alexa 488-labeled RNAs or PBS (vehicle control).

**
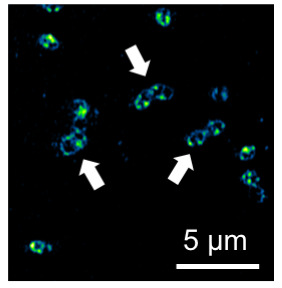
**

**Figure S13:** Periphery localization of tsRNA-000794-Cy3 in *S. mitis* ATCC 6249 revealed by Airyscan confocal microscopy. 128 nM tsRNAs were incubated with bacteria overnight under anaerobic conditions.


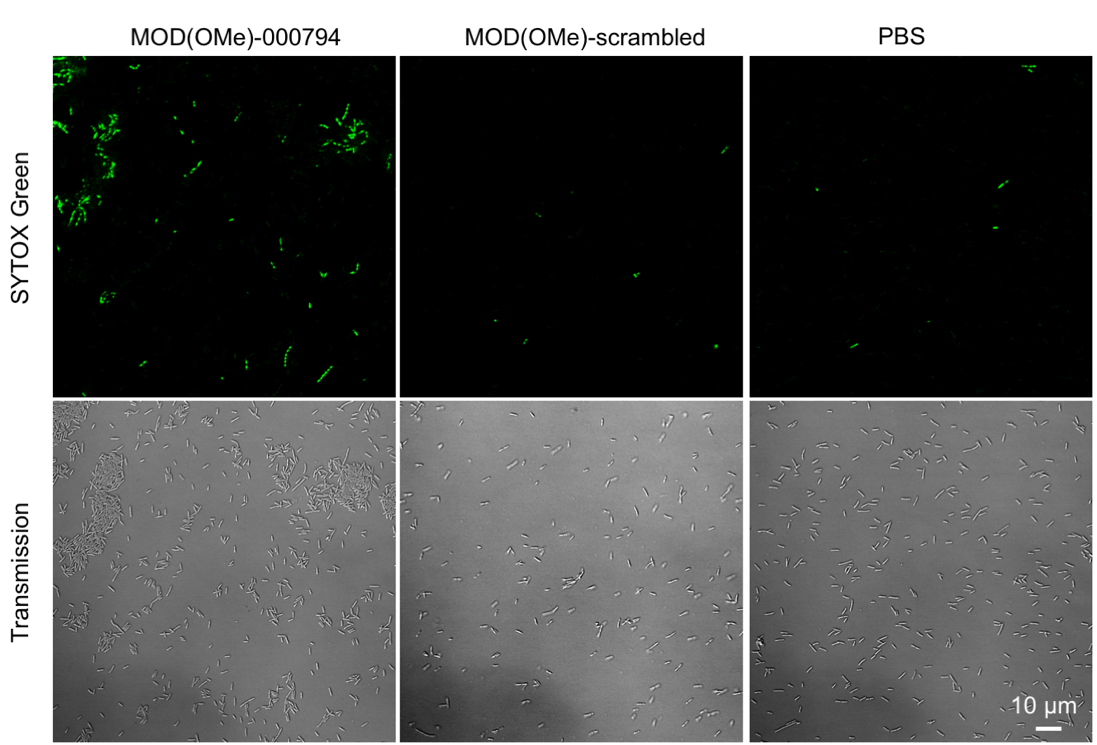


**Figure S14:** SYTOX green viability examination of *Fn* ATCC 23726 after treatment with 500 nM MOD(OMe)-000794, MOD(OMe)-scrambled RNA and 1xPBS for 5 h before harvesting for RNA sequencing or Raman microscopy. Bacteria were treated at a starting OD_600_ of 0.2. Images are representative of three biological replicates.


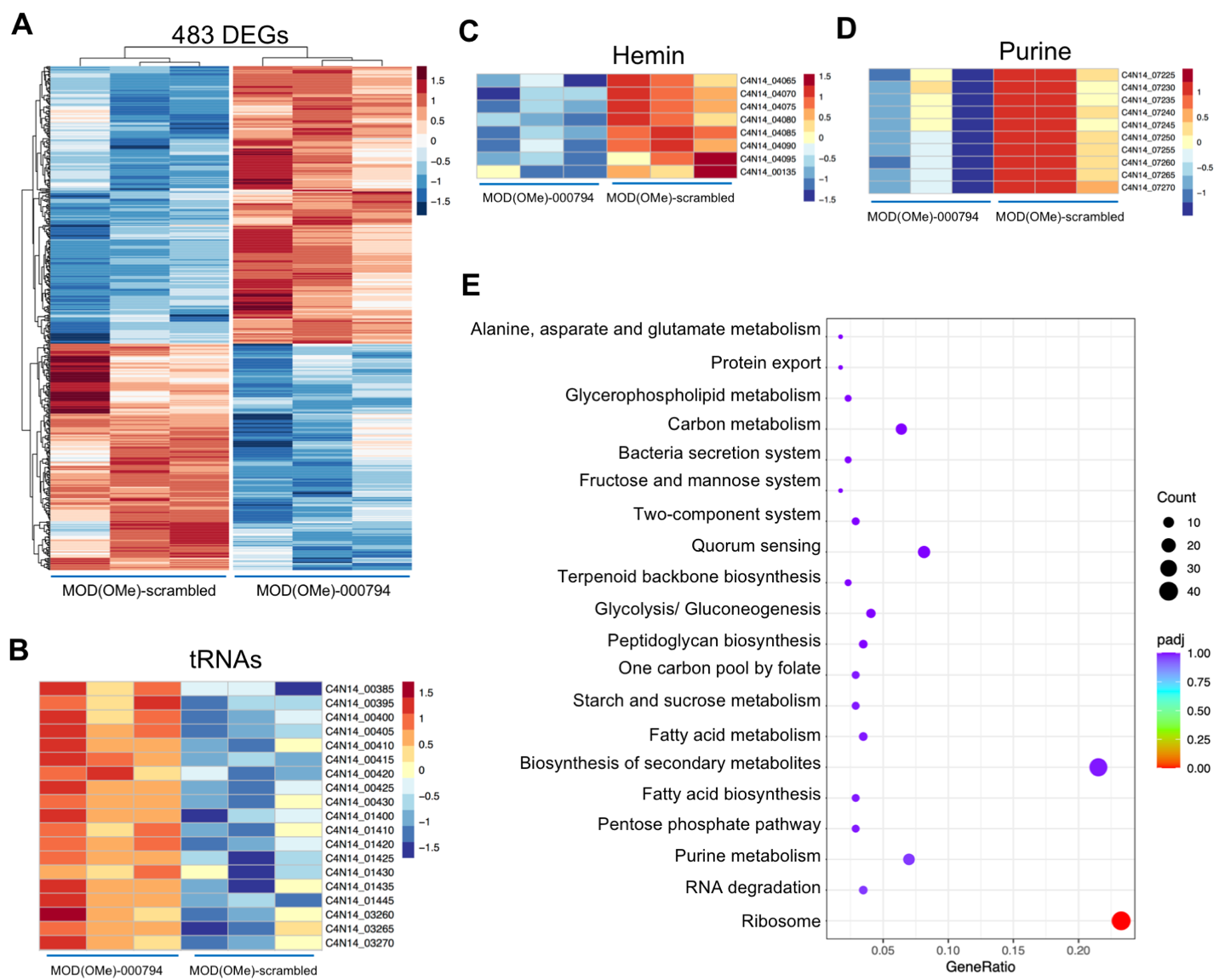


**Figure S15:** (*A*) Cluster analysis showing 483 differentially expressed genes (DEGs) using the DESeq2 method (*p*-adj<=0.05, |log2FoldChange|>=0.0). (*B*) genes encoding tRNAs. (*C*) genes encoding hemin intake function, and (*D*) genes encoding function involved in purine biosynthesis are presented in the heatmaps. Each heatmap includes triplicate RNA-seq samples for the indicated MOD(OMe)-000794 or MOD(OMe)-scrambled treatment (5 h, 500 nM). The coloring indicates log_2_FoldChange (FC) of the selected samples, while red and blue denote up- and down-regulation, respectively. (*E*) KEGG enrichment scatter plot of DEGs. The y-axis shows the name of the pathway, and the x-axis shows the Rich factor. Dot size represents the number of different genes, and the color indicates the *p*-value adjusted (*p*-adj).


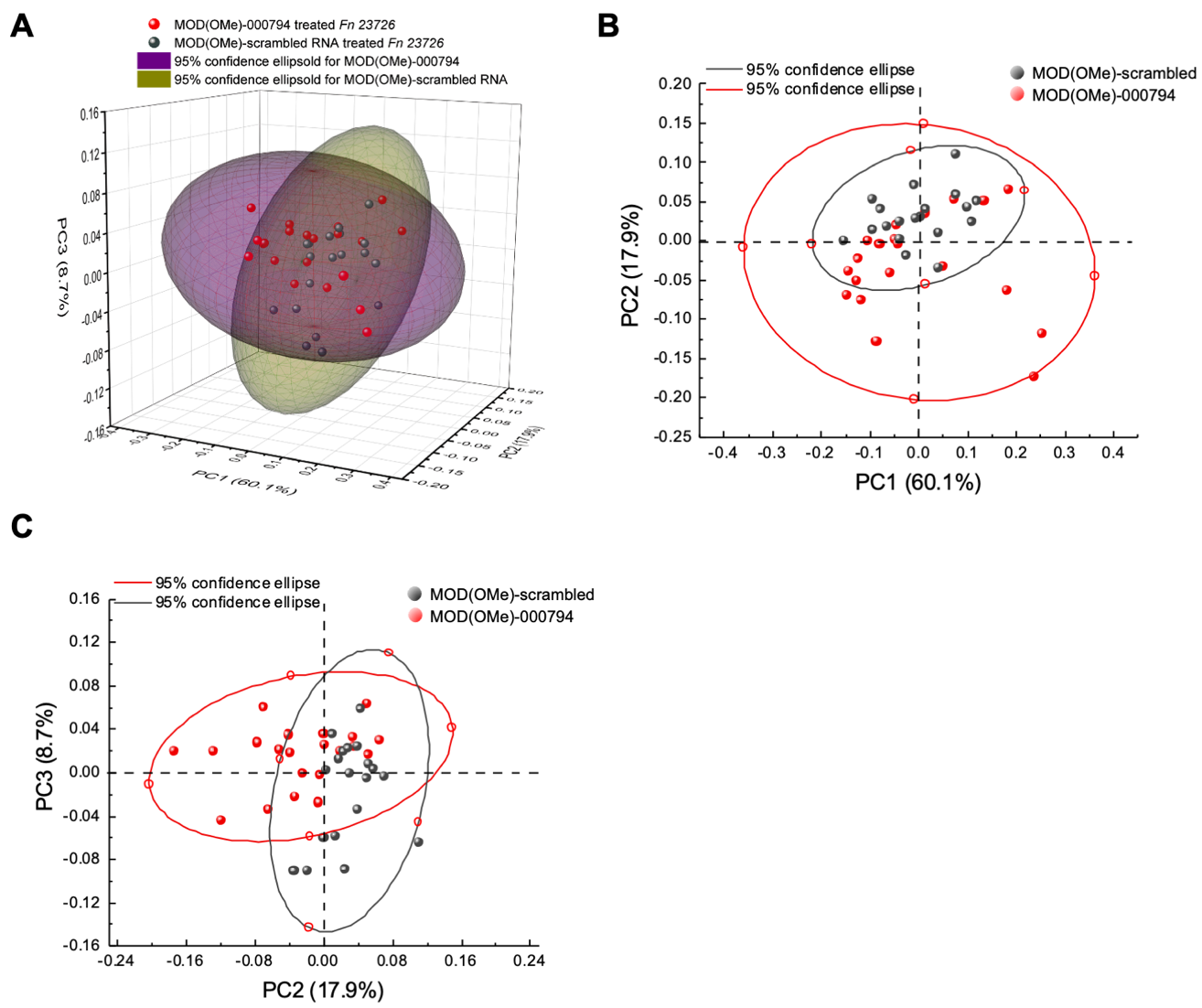


**Figure S16:** (*A*) Principal component analysis (PCA) of Raman spectra of *Fn* ATCC 23726 after treatment with 500 nM MOD(OMe)-000794, MOD(OMe)-scrambled RNA for 5 h. Three-dimensional PCA plot indicates global difference as to the Raman spectra from MOD(OMe)-000794 and scrambled RNA treated *Fn* ATCC 23726. Three-dimensional ellipse represents the 95% confidence interval. (*B* and *C*) three two-dimensional PCA plots suggests PC3 contributes to the most difference between MOD(OMe)-000794 and -scrambled RNA treated *Fn* ATCC 23726. Each dot represents a single Raman spectrum from *Fn* ATCC 23726 aggregates dried on the aluminum substrate. Two-dimensional ellipse represents the 95% confidence interval line.

**Table S1.** A list of representative DEGs identified from RNAseq.

| **Locus Tag** | **Log_2_FoldChange^1^** | ***p*-adj** | **Gene Description** |
| --- | --- | --- | --- |
| C4N14_01875 | 1.23 | 8.36E-14 | 50S ribosomal protein L1 |
| C4N14_00690 | 1.37 | 3.57E-16 | 50S ribosomal protein L30 |
| C4N14_00410 | 1.64 | 1.93E-05 | tRNA-Met |
| C4N14_03265 | 0.93 | 0.036104 | tRNA-Lys |
| C4N14_01710 | 2.04 | 7.07E-23 | Chaperone DnaJ |
| C4N14_01725 | 2.31 | 8.77E-18 | Chaperone DnaK |
| C4N14_08865 | 1.70 | 7.17E-13 | Chaperonin GroEL |
| C4N14_03355 | 2.82 | 3.37E-50 | ATP-dependent chaperone ClpB |
| C4N14_07245 | -1.74 | 0.000470171 | Phosphoribosylformylglycinamidine cyclo-ligase |
| C4N14_07270 | -1.95 | 3.54E-06 | Phosphoribosylamine--glycine ligase |
| C4N14_07225 | -1.26 | 0.00078033 | Phosphoribosylformylglycinamidine synthase |
| C4N14_07230 | -1.35 | 0.01388648 | 5-(carboxyamino)imidazole ribonucleotide mutase |
| C4N14_04075 | -1.80 | 1.83E-10 | Hemin transporter |

^1^Fold changes by RNAseq reflect gene expression of the treatment group 500 nM MOD(OMe)-000794 relative to MOD(OMe)-scrambled RNA control.

**Table S2**. Primer sequences used for RNAseq verification.

| Primer name | Locus Tag | Sequence (5'-3') |
| --- | --- | --- |
| Q176 | 16S | Fwd: CTTAGGAATGAGACAGAGATG |
| Q177 |  | Rev: TGATGGTAACATACGAAAGG |
| Q273 | C4N14_01875 | Fwd: AGATCCAAGACATGCTGACCA |
| Q274 |  | Rev: TCTGCTCCTGCAGCTAATGC |
| Q279 | C4N14_00410 | Fwd: GCTCAGCTGGATAGAGCAACGC |
| Q280 |  | Rev: GGATCCAGCTGGACTCGAACCA |
| Q281 | C4N14_01710 | Fwd: TGGTGGCTTCAATGCAGGAG |
| Q282 |  | Rev: AAACCTCCAAAGCCTCCACC |
| Q283 | C4N14_07245 | Fwd: AACTGCTGAAATGCCAGGCT |
| Q284 |  | Rev: ATCCACTTGAAGCCACTGCT |
| Q289 | C4N14_07270 | Fwd: GAGAATGGGCGACCCTGAAA |
| Q290 |  | Rev: ATCCACCTGCTGCCATAACC |
| Q291 |  | Fwd: CGGCAGGAAAGGGTGTTGTA |
| Q292 |  | Rev: TCTCCTGCAGCAGCAAATACT |
| Q297 | C4N14_07225 | Fwd: GCAGCACCTGTGGAAAATGT |
| Q298 |  | Rev: GAACCAGTTGCTCCTCCACA |
| Q299 | C4N14_07230 | Fwd: AAGGGAGCAGCAGACTGTTT |
| Q300 |  | Rev: AATGTGCTGCAAGTCCTGCT |
| Q301 | C4N14_01725 | Fwd: GCTGCTGCTCTTGCTTATGG |
| Q302 |  | Rev: AATGTTCCCCCACCAAGGTC |
| Q303 |  | Fwd: CCAGCAGTTCAAGAATGGGT |
| Q304 |  | Rev: CTTGTATTGCAGCACCTGCC |
| Q305 | C4N14_08865 | Fwd: TGAAAATATGGGGGCAGCCTT |
| Q306 |  | Rev: TGTTGTTCCGTCTCCTGCAA |
| Q311 | C4N14_03355 | Fwd:TTGGTGAACCTGGAGTTGGT |
| Q312 |  | Rev: CCTGCAACCAAAGCTCCCAT |
| Q313 |  | Fwd: GCCGTTAAATCCGTTGCTGA |
| Q314 |  | Rev: TTACCAACCCCAGTAGGTCC |
| Q315 | C4N14_03265 | Fwd: CGACCATTAGAGTGCGGGAA |
| Q316 |  | Rev: CGACAACCAGTGCCACAAAC |
| Q319 | C4N14_00690 | Fwd: TGCAGGTAAATGGGGAGCAA |
| Q320 |  | Rev: GCAGAACCAGCTATTACCCCA |
| Q323 | C4N14_04075 | Fwd: TGATGCCTTAAGAGCGAAAGGT |
| Q324 |  | Rev: TGGTCTCTATTTGCACTTGCT |
| Q325 |  | Fwd: TGCATTAGGACATGAGTTTGGACA |
| Q326 |  | Rev: TGCTGCTAAGGTATCTTCTGTTGC |

**Table S3**. The Stem-loop primer sequences used for MOD-tsRNA stability test.

| Q169 | 020498 stem loop | GTCGTATCCAGTGCAGGGTCCGAGGTATTCGCACTGGATACGACATGCTCTA |
| --- | --- | --- |
| Q189 | 000794 stem loop | GTCGTATCCAGTGCAGGGTCCGAGGTATTCGCACTGGATACGACTCTCATGC |
| Q190 | Scrambled stem loop | GTCGTATCCAGTGCAGGGTCCGAGGTATTCGCACTGGATACGACCAGATGCG |
| Q184 | 000794 qPCR Fwd | AGACCGGCTAGCTCAGT |
| Q185 | 020498 qPCR Fwd | AAGGGGGGTATAGCTCAGT |
| Q187 | Scrambled qPCR Fwd | AAGGGACGACAAGTTCGTGACG |
| Q167 | tsRNA qPCR Rev | GTCGTATCCAGTGCAGGGT |

**Table S4**. Statistical analyses for RT-PCR verification of RNAseq. Statistical significance was determined by the paired t-test after normalization to 16s. * *p* ≤ 0.05, ** *p* ≤ 0.01.

| Locus Tag | Log_2_FoldChange | *p*-value | Symbols |
| --- | --- | --- | --- |
| C4N14_01875 | 1.55 | 0.1862 | ns |
| C4N14_00690 | 1.47 | 0.1967 | ns |
| C4N14_00410 | 0.70 | 0.5490 | ns |
| C4N14_03265 | 0.71 | 0.2123 | ns |
| C4N14_01710 | 2.36 | 0.0206 | * |
| C4N14_01725 | 2.65 | 0.0104 | * |
| C4N14_08865 | 1.85 | 0.0474 | * |
| C4N14_03355 | \| 4.00 \| \| --- \| | \| 0.0010 \| \| --- \| | * |
| C4N14_07245 | -1.35 | 0.2312 | ns |
| C4N14_07270 | -1.76 | 0.0940 | ns |
| C4N14_07225 | -0.96 | 0.9378 | ns |
| C4N14_07230 | \| -1.03 \| \| --- \| | 0.8937 | ns |
| C4N14_04075 | -1.30 | 0.2568 | ns |

**Table S5**. Statistical analyses for RT-PCR of 1 μg mL^-1^ thiamphenicol treated *Fn*. Statistical significance was determined by the paired t-test after normalization to 16s. **p* ≤ 0.05, ***p* ≤ 0.01.

| Locus Tag | Log2FoldChange | *p* -value | Symbols |
| --- | --- | --- | --- |
| C4N14_01875 | 3.54 | 0.0089 | ** |
| C4N14_00690 | 1.78 | 0.0420 | * |
| C4N14_00410 | 3.36 | 0.0157 | * |
| C4N14_03265 | 3.35 | 0.0030 | ** |
| C4N14_01710 | 1.38 | 0.0439 | * |
| C4N14_01725 | 1.30 | 0.3290 | ns |
| C4N14_08865 | 0.25 | 0.0277 | * |
| C4N14_03355 | 0.31 | 0.0200 | * |
| C4N14_07245 | 0.48 | 0.1048 | ns |
| C4N14_07270 | 0.63 | 0.0613 | ns |
| C4N14_07225 | 0.55 | 0.1085 | ns |
| C4N14_07230 | 1.35 | 0.3360 | ns |
| C4N14_04075 | -1.02 | 0.9372 | ns |
